# Fungal ITS1 Deep-Sequencing Strategies to Reconstruct the Composition of a 26-Species Community and Evaluation of the Gut Mycobiota of Healthy Japanese Individuals

**DOI:** 10.3389/fmicb.2017.00238

**Published:** 2017-02-15

**Authors:** Daisuke Motooka, Kosuke Fujimoto, Reiko Tanaka, Takashi Yaguchi, Kazuyoshi Gotoh, Yuichi Maeda, Yoki Furuta, Takashi Kurakawa, Naohisa Goto, Teruo Yasunaga, Masashi Narazaki, Atsushi Kumanogoh, Toshihiro Horii, Tetsuya Iida, Kiyoshi Takeda, Shota Nakamura

**Affiliations:** ^1^Department of Infection Metagenomics, Genome Information Research Center, Research Institute for Microbial Diseases, Osaka UniversitySuita, Japan; ^2^Laboratory of Immune Regulation, Department of Microbiology and Immunology, Graduate School of Medicine, WPI Immunology Frontier Research Center, Osaka UniversitySuita, Japan; ^3^Department of Respiratory Medicine, Allergy and Rheumatic Diseases, Graduate School of Medicine, Osaka UniversitySuita, Japan; ^4^Division of Bio-resources, Medical Mycology Research Center, Chiba UniversityChiba, Japan; ^5^Department of Bacteriology, Graduate School of Medicine, Dentistry and Pharmaceutical Sciences, Okayama UniversityOkayama, Japan

**Keywords:** mycobiota, fungi, internal transcribed spacer (ITS), high-throughtput sequencing (HTS), the gut mycobiota in Japanese

## Abstract

The study of mycobiota remains relatively unexplored due to the lack of sufficient available reference strains and databases compared to those of bacterial microbiome studies. Deep sequencing of Internal Transcribed Spacer (ITS) regions is the *de facto* standard for fungal diversity analysis. However, results are often biased because of the wide variety of sequence lengths in the ITS regions and the complexity of high-throughput sequencing (HTS) technologies. In this study, a curated ITS database, ntF-ITS1, was constructed. This database can be utilized for the taxonomic assignment of fungal community members. We evaluated the efficacy of strategies for mycobiome analysis by using this database and characterizing a mock fungal community consisting of 26 species representing 15 genera using ITS1 sequencing with three HTS platforms: Illumina MiSeq (MiSeq), Ion Torrent Personal Genome Machine (IonPGM), and Pacific Biosciences (PacBio). Our evaluation demonstrated that PacBio’s circular consensus sequencing with greater than 8 full-passes most accurately reconstructed the composition of the mock community. Using this strategy for deep-sequencing analysis of the gut mycobiota in healthy Japanese individuals revealed two major mycobiota types: a single-species type composed of *Candida albicans* or *Saccharomyces cerevisiae* and a multi-species type. In this study, we proposed the best possible processing strategies for the three sequencing platforms, of which, the PacBio platform allowed for the most accurate estimation of the fungal community. The database and methodology described here provide critical tools for the emerging field of mycobiome studies.

## Introduction

Comprehensive analysis of bacterial communities (microbiota) has been made possible with the advent of high-throughput sequencing (HTS) technologies. Particularly, this has led to understanding the composition of the gut microbiome as influenced by factors, such as genetic background, diet, and immune function. Rapid progress has been made in discovery of associations between gut microbiota, the host’s immune system, and various diseases (Arumugam et al., 2011; Wu et al., 2011; Gevers et al., 2012; Ursell et al., 2012).

In addition to bacteria, various types of fungi are also present in the intestines (Schulze and Sonnenborn, 2009; Chen et al., 2011; Dollive et al., 2012). Although the absolute number of commensal fungi accounts for as little as approximately 0.1% of total microorganisms in the intestines (Underhill and Iliev, 2014), they may have an important role in human health (Chang et al., 2011; Gaitanis et al., 2012; Cui et al., 2013; Huffnagle and Noverr, 2013; Wang et al., 2014). For instance, *Candida albicans* is a species of fungi persistently present in model mice that causes the exacerbation of allergies and autoimmune diseases (Sonoyama et al., 2011). In addition, fungi in the intestines induce colitis through the activity of host Dectin-1, a C-type lectin receptor (Sonoyama et al., 2011; Iliev et al., 2012). Fungal communities (mycobiota) in the intestines clearly play an important role in the health of the host.

Various genera of fungi have been detected in mycobiota analyses of the healthy human gut, and the genera *Saccharomyces. Candida*, and *Cladosporium* have been detected in particularly high percentages (Schulze and Sonnenborn, 2009; Chen et al., 2011; Dollive et al., 2012; Hoffmann et al., 2013). Moreover, analyses of the mycobiota in the oral cavity (Kleinegger et al., 1996; Ghannoum et al., 2010) and skin (Paulino et al., 2006, 2008; Zhang et al., 2011; Park et al., 2012; Findley et al., 2013) have demonstrated that mycobiota populations vary according to sites on the body (Cui et al., 2013; Underhill and Iliev, 2014). Furthermore, there have recently been a number of discussions on the relationships between both fungi and host and among bacteria, fungi, and host (Romani and Luigina, 2011; Mason et al., 2012a,b; Huffnagle and Noverr, 2013; Underhill and Iliev, 2014; Wang et al., 2014; Romani et al., 2015). However, unlike studies analyzing the bacterial component of the host microbiome, fewer studies are available on mycobiomes. In addition, databases of fungal sequences and methodologies for mycobiota analysis are not yet fully developed. Therefore, the potential impact of the mycobiome on pathogenesis and the development of the host immune system remains unknown (Romani and Luigina, 2011; Cui et al., 2013; Huffnagle and Noverr, 2013; Underhill and Iliev, 2014; Wang et al., 2014).

Characterizations of mycobiomes mainly rely on analysis of one portion of the internal transcribed spacer (ITS) region, ITS1, which is located between the 18S and 5.8S subunits of the fungal rRNA genes (Huffnagle and Noverr, 2013; Underhill and Iliev, 2014). This region is universally present in fungi and the mutation rate has been used to analyze evolutionary relationships. For these reasons, it is widely used for identification of species and genetic analyses. However, while the 16S rRNA gene used for bacterial microbiota analysis has a virtually identical length regardless of the species, the length of the ITS region in fungi varies between species. MiSeq and IonPGM sequencing platforms commonly used in mycobiota analysis are able to produce read lengths of several hundred base pairs (bp), however, shorter DNA sequences are favored on these platforms, and thus, overrepresented in the sequencing results. For example, the ITS1 sequence of the well-known fungi *Saccharomyces cerevisiae* has a length of approximately 460 bp, however, IonPGM do not completely read the ITS1 region with sufficient margins. As a result, it is impossible to have an accurate census of the composition of some fungi in the mycobiota using this strategy. A previous study aimed at solving this issue made attempts to evaluate the accuracy of the analysis by using the ITS1 sequence together with additional gene regions (Tonge et al., 2014). In addition to the problem of short reads, there is also a challenge in establishing a consensus method for mycobiome assessment. There are studies approached with the analytical strategy using automated analysis platforms such as QIIME (Caporaso et al., 2010) and PIPITS (Gweon et al., 2015), and using some curated databases of fungal ITS sequences (Findley et al., 2013; Kõljalg et al., 2013; Schoch et al., 2014; Tang et al., 2015). However, due to the complexity in evaluating the combination of both experimental and analytical strategies, not many attempts have been made to establish a consensus method for mycobiome assessment.

In this study, combinations of experimental and analytical strategies using three sequencing technologies, Illumina MiSeq (MiSeq), Life Technologies Ion Torrent PGM (IonPGM), and Pacific Biosciences RS II system (PacBio), were evaluated using a newly constructed database, ntF-ITS1, and a mock community containing mixtures of 26 fungal species representing 15 genera. Because reports of the analysis of human mycobiomes have been relatively rare and the gut mycobiota is particularly poorly understood (Cui et al., 2013; Huffnagle and Noverr, 2013; Tonge et al., 2014; Wang et al., 2014), we analyzed the mycobiota of feces from healthy Japanese subjects. In this paper, we propose the best strategies to assess the fungal communities and mycobiota of healthy Japanese individuals that is a potential consensus method for mycobiome assessment that may overcome the problem of short reads.

## Materials and Methods

### Database Construction

Sequences classified as fungi (Taxonomy ID:4751) were extracted from the NCBI-nt database. Additional sequences with ambiguous taxonomic information (those whose taxonomic name included the terms “uncultured,” “environmental,” “unclassified,” or “mixed”) were excluded. From these sequences, the ITS1 region was identified between the ITS1-F (5′-CTTGGTCATTTAGAGGAAGTAA-3′) and ITS2 (5′-GCATCGATGAAGAACGCAGC-3′) sequences using cutadapt-1.7.1 with default parameters (Martin and Marcel, 2011). Because no ITS1 sequences were identified for *Rhodosporidium babjevae. Acremonium alternatum. Penicillium digitatum. Mucor ramosissimus*, or *Rhizopus oryzae* in the curated NCBI-nt database, we manually added these sequences to our database. The resulting sequences were converted into a format that was suitable for the Ribosomal Database Project (RDP) Classifier (Wang et al., 2007), and this was used as the database nt-Fungi-ITS1 (ntF-ITS1) for mycobiota analysis. Sequence extraction and format conversion were conducted with a Ruby script. The ntF-ITS1 database can be downloaded from our website^1^.

### Culture and DNA Extraction from 26 Known Fungal Species

The 26 examined strains listed in **Table 1** were cultivated on potato-dextrose agar (PDA) slants at 25°C for 3 – 7 days. DNA was extracted from each of cultivated mycelium using a modified benzyl bromide extraction method reported previously in Zhu et al. (1993). Briefly, 500 μL of extraction buffer (100 mM Tris-HCl, 40 mM EDTA, pH 9.0), 100 μL of 10% SDS, and 300 μL of benzyl chloride were added to approximately 200 mg of mycelium. This suspension was then mixed by vortexing and incubated at 65°C for 25 min. The mixture was kept on ice for 5 min and centrifuged at 6,000 × *g* for 10 min at 4°C. DNA was collected from the supernatant by isopropanol precipitation.

**Table 1:** Genera, species, ITS1 length, and the percentage of fungi used in the mock community.

Fungi	IFM^a^	genus	species	ITS1 length/bp	Percentage of each species in the mock community^b^
1	61908	*Acremonium*	*Acremonium alternatum*	274	4.1%
2	61916	*Aspergillus*	*Aspergillus flavus*	299	3.7%
3	54229	*Aspergillus*	*Aspergillus fumigatus*	302	3.7%
4	62238	*Aspergillus*	*Aspergillus niger*	327	3.4%
5	61604	*Aspergillus*	*Aspergillus terreus*	304	3.7%
6	40009	*Candida*	*Candida albicans*	256	4.3%
7	48313	*Candida*	*Candida dubliniensis*	258	4.3%
8	61949	*Candida*	*Candida tropicalis*	279	4.0%
9	62110	*Cladosporium*	*Cladosporium cladosporioides*	273	4.1%
10	57770	*Cladosporium*	*Cladosporium herbarum*	272	4.1%
11	50258	*Cryptococcus*	*Cryptococcus aureus*	228	4.9%
12	46660	*Filobasidiella*	*Cryptococcus neoformans*	239	4.7%
13	62224	*Fusarium*	*Fusarium oxysporum*	265	4.2%
14	62065	*Fusarium*	*Fusarium solani*	267	4.2%
15	47659	*Mucor*	*Mucor ramosissimus*	326	3.4%
16	5768	*Nakaseomyces*	*Candida glabrata*	520	2.1%
17	61632	*Penicillium*	*Penicillium chrysogenum*	293	3.8%
18	62178	*Penicillium*	*Penicillium citrinum*	257	4.3%
19	60598	*Penicillium*	*Penicillium digitatum*	292	3.8%
20	49446	*Penicillium*	*Penicillium oxalicum*	294	3.8%
21	47055	*Rhizopus*	*Rhizopus oryzae*	310	3.6%
22	48570	*Rhodosporidium*	*Rhodosporidium babjevae*	270	4.1%
23	40060	*Rhodotorula*	*Rhodotorula mucilaginosa*	270	4.1%
24	40022	*Saccharomyces*	*Saccharomyces cerevisiae*	483	2.3%
25	51186	*Trichoderma*	*Trichoderma viride*	301	3.7%
26	51050	*Trichoderma*	*Trichoderma koningii*	301	3.7%


*^a^IFM is the abbreviation for Institute of Food Microbiology. ^b^Each percentage is the theoretical mock community abundance of each fungal species. The amplicons were mixed to yield equal amount of DNA (each was 50 ng) of each sample.*

### Amplification of ITS1 Regions and Sanger Sequencing of the Fungal Mock Community

PCR was performed using a primer set (ITS1-F: 5**′**-CTTGGTCATTTAGAGGAAGTAA-3**′**) and (ITS2: 5**′**-GCATCGATGAAGAACGCAGC-3**′**) targeted the ITS1 region of the ITS. To amplify the targeted region, 5 ng of extracted DNA from 26 known individual fungal species served as the template in 50 μL reactions using KAPA HiFi HotStart Ready Mix (KAPA Biosystems, Woburn, MA, USA). DNA was amplified with an initial denaturation step at 95°C for 3 min, followed by 15 cycles of denaturation at 98°C for 20 s, annealing at 56°C for 15 s, and elongation at 72°C for 30 s. Products were purified using DNA clean and Concentrator-5 (Zymo Research, Orange, CA, USA). Sequencing reactions were performed using 3130xI Genetic Analyzer (Applied Biosystems, Foster City, CA, USA).

### Library Preparation, Sequencing, and Read Trimming for the Fungal Mock Community

The concentrations of the ITS1 amplicons from all 26 fungal species were measured using a Qubit Fluorometer (Invitrogen, Carlsbad, CA, USA). Amplicons were mixed to yield equal amounts of DNA (50 ng each), and the mixture was used as a fungal mock community for the preparation of libraries for each sequencing system. Since different fungal species has different ITS1 length, percent composition of each fungi in the mock community based on their ITS1 length is calculated as shown in the **Table 1**. The raw sequencing data have been deposited in the DDBJ Sequence Read Archive (DRA) under the accession code DRA004340.

### IonPGM

A library was prepared from 5 ng of mixed amplicon using the Ion Fragment Library kit (Life Technologies, Gaithersburg, MD, USA) according to the manufacturer’s instructions. Sequencing was performed using a 318 chip and Ion PGM Sequencing Hi-Q Kit (Life Technologies). Raw sequences were trimmed using BBtrim software^2^ with mean quality value of 10–30. Sequences without full length ITS1 (i.e., the sequences amplified with primers by PCR) were removed using the FASTX-Toolkit^3^ for subsequent analysis.

### MiSeq

A library was prepared from 10 ng of mixed amplicon using KAPA Library Preparation kits (KAPA Biosystems) according to the manufacturer’s instructions. Paired-end sequencing of 251 bp was performed using a MiSeq v2 500 cycle kit (Illumina, San Diego, CA, USA). Paired-end sequences were merged using PEAR software^4^. The merged reads were then quality filtered with the same condition as those of the IonPGM data.

### PacBio

A library was prepared from 300 ng of mixed amplicon using the DNA Template Prep kit 2.0 (Pacific Biosciences, Menlo Park, CA, USA) according to the manufacturer’s instructions. Sequencing was performed with the PacBio RS II system using the DNA Sequencing Kit C2 (Pacific Biosciences) with P4 polymerase. CCS constructed from more than three full-pass subreads were produced using PacBio SMRT Analysis.

### Fungal ITS1 Deep Sequencing of Healthy Japanese Feces

Feces were collected in tubes containing *RNAlater* (Ambion, Austin, TX, USA). Samples were weighed, and *RNAlater* added to make 10-fold dilutions of homogenates. Homogenates (200 mg) of feces were washed twice with 1 mL PBS and fecal DNA was extracted with the PowerSoil DNA isolation kit (MO BIO Laboratories, Solana Beach, CA, USA) according to the manufacturer’s protocol. Samples were stored at -20°C. DNA was amplified with PCR using the following protocol: Initial denaturation at 95°C for 2 min, followed by 40 cycles of denaturation at 95°C for 20 s, annealing at 56°C for 30 s, elongation at 72°C for 30 min, followed by a final elongation step at 72°C for 10 min. Barcoded PacBio libraries were prepared using the DNA Template Prep kit 2.0 (Pacific Biosciences, Menlo Park, CA, USA) according to the manufacturer’s instructions. Three libraries per SMRT Cell were pooled and subjected to Single Molecule Real-Time (SMRT) sequencing using the PacBio RS II system (Pacific Biosciences). All fecal samples were collected and analyzed at Osaka University. The Osaka University ethics committee approved this study and written informed consent was obtained from all study subjects (12237-3).

### Bioinformatic Analysis and Taxonomic Assignment

Sequences were clustered into operational taxonomic units (OTUs), defined at 90 – 100% similarity cutoff for the mock community and 95% for fecal samples using UCLUST version 1.2.22q (Edgar, 2010) using the script (pick_otus.py) in QIIME 1.9.1 (pick_otus.py). Representative sequences for each OTU were classified taxonomically using RDP Classifier version 2.2 using the script (assign_taxonomy.py) in QIIME 1.9.1 with our ntF-ITS1 database and the minimum confidence value is 0.55, and using blastn 2.3.30+ using the script (assign_taxonomy.py) in QIIME 1.9.1 with the default parameters. For the analysis of mock community, we also used the databases; UNITE (Kõljalg et al., 2013), Findley (Findley et al., 2013), and THF (Tang et al., 2015). The characterization of these ITS reference database were summarized in **Supplementary Table S1**. For principal component analysis (PCA), we visualized data sets using the statistical programming language R 3.1.3 (R Core Team, 2016). Hierarchical clusters of mycobiota were calculated from the relative abundances and the ranked abundances of genera. PCA was performed using the “stats” package from CRAN. A heat map visualization of OTUs was generated with the heatmap.2 function of the “gplots” package from CRAN.

## Results

### Construction of a Fungal ITS1 Database

**FIGURE 1:**
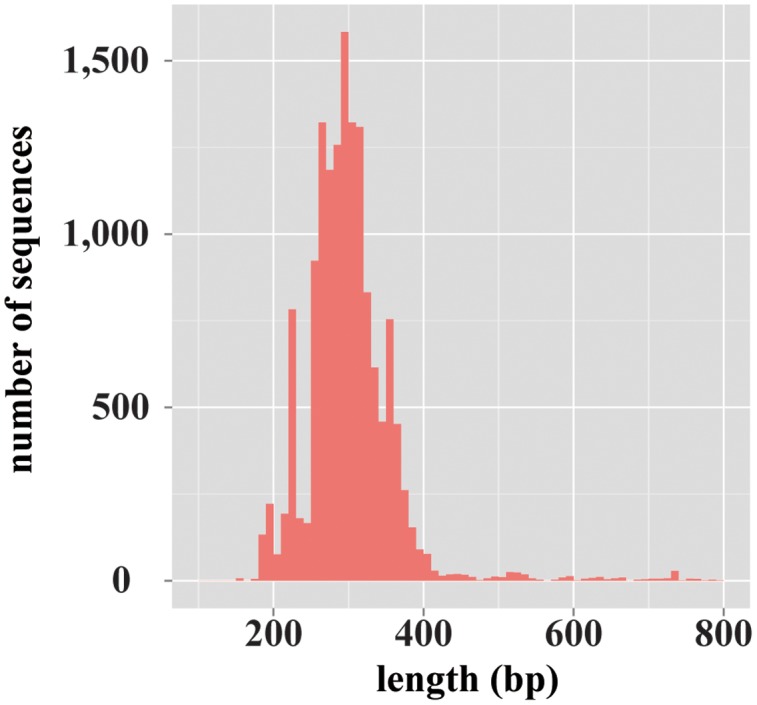
**The length distribution of the fungal ITS1 region sequences registered on ntF-ITS1 database.** A histogram showing the variety in length distribution of fungal ITS1 region sequences. The horizontal axis represents the length of ITS1 sequences. The vertical axis represents the number of sequences of each length.

For the database construction to identify fungal species, at first, 2,444,619 records of fungi-derived sequences were retrieved from the NCBI-nt database. Of those records, 1,849,386 sequences remained after excluding the records that included ambiguous taxonomic information (e.g., “environmental samples”). Finally, from these, 13,943 sequences remained by excluding the data records with no ITS1 region information, and the database has been constructed. The constructed database, termed ntF-ITS1, represented 1,218 genera and 6,525 species. The average length of ntF-ITS1 sequences was 257 bp, and was widely distributed from approximately 100–800 bp (**Figure 1**). The length of ITS1 for each sequence in THF and UNITE showed the similar distribution to ntF-ITS1 as shown in **Supplementary Figure S1**. The number of fungal genera and species included in each database were summarized in **Supplementary Table S1**.

### Taxonomic Assignment of a Mock Community

A mock community composed of 26 fungal species representing 15 genera (**Table 1**) was used to evaluate the various methods of taxonomic assessment. To characterize this community, we sequenced the ITS1 regions of 26 species. The average length of ITS1 in the mock community was 298 bp; the shortest sequence measured was 228 bp, and the longest 520 bp. Thus, the distribution of ITS1 sequence lengths in the mock community were of similar diversity to ntF-ITS1. We next examined the percent identity that allowed for accurate OTU clustering of the mock community. Using a similarity threshold of 98% or higher for clustering, the 26 fungal species were classified as 25 individual OTUs. *Trichoderma viride* and *Trichoderma koningii* were clustered in the same OTU due to the fact that their sequences had 100% similarity. Using a threshold of 97% similarity, *Penicillium digitatum* and *Penicillium chrysogenum* were classified in the same OTU. Using a threshold of 96% similarity, *Cladosporium herbarum* and *Cladosporium cladosporioides* were classified in the same OTU. At a 95% similarity threshold, *C. albicans* and *C. dubliniensis* were classified in the same OTU. If the similarity threshold were reduced to 94% and below, fungal species from differing genera (*Penicillium chrysogenum* and *Aspergillus fumigatus*) were clustered in the same OTU. These clustering results at each similarity level are summarized in **Supplementary Table S2**. We also classified each OTU using blastn and UCLUST. As shown in **Supplementary Table S3**, blastn and UCLUST mis-assigned some OTU’s to other genera. Our evaluation suggested that to conduct a clustering of fungal genera on the basis of the ITS1 region in an accurate manner, the sequence similarity required for clustering must be 95% or higher. We employed the 95% sequence similarity threshold for taxonomic assignment with our ntF-ITS1 using RDP Classifier, resulting in the accurate assignments of all 15 fungal genera in our mock community (**Supplementary Table S4**). We further compared our ntF-ITS1 to existing fungal ITS database, UNITE, Findley, and THF. As shown in the **Supplementary Table S5**, while some sequences were mis-assigned to wrong genera using UNITE and Findley, all sequences were assigned to correct genera using ntF-ITS1 and THF. While THF has sequences for only 1,816 species, ntF-ITS1 for 6,525 species. Therefore we used ntF-ITS1 for analysis of fungal ITS1 deep sequence.

### Mock Community Analysis by IonPGM Sequencing

**FIGURE 2:**
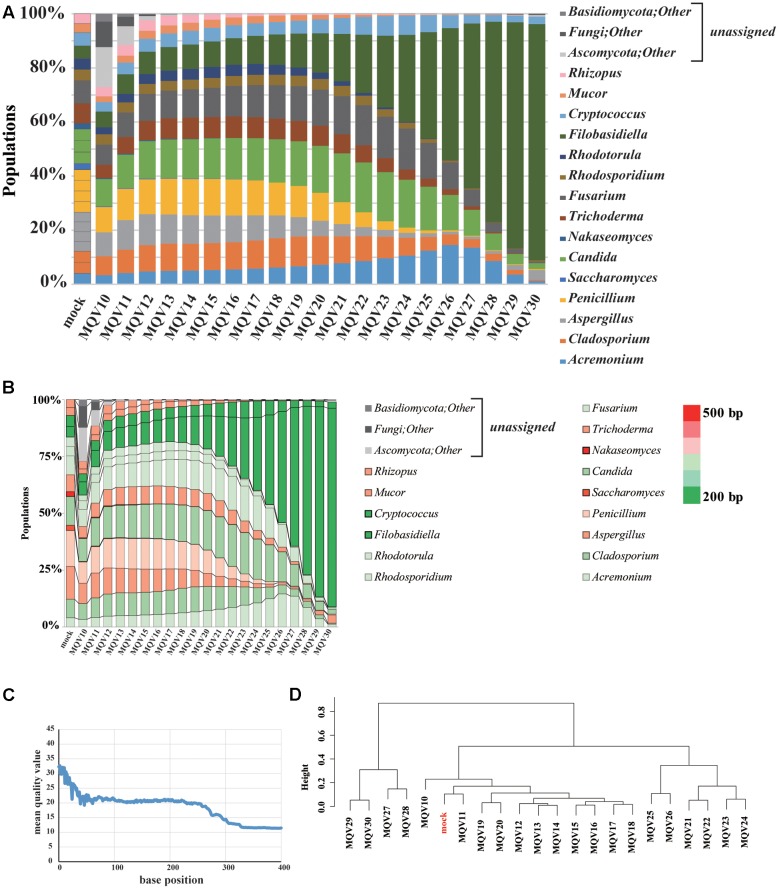
**Results of mycobiota analysis in the mock community using IonPGM sequencing.** **(A)** Relative abundance of major fungal genera in the mock community shown in a bar graph. Read-trimming was carried out using an ideal relative abundance of the mock community and MQV 10 – 30. Percentage read abundances of 15 genera are shown in **Supplementary Figure S4**. **(B)** Relative abundances of the major fungal genera are shown in different colors according to the length of the ITS1 region. **(C)** MQV at each base position of the read. **(D)** Genus-level hierarchical clustering analysis of the relative abundance of the major fungal genera. Results of analyses conducted using MQV ranging from 10 to 30 were analyzed along with ideal values.

The Ion PGM Hi-Q sequencing of the mock community yielded 219,756 reads. We evaluated the composition of the community with trimming conditions at varying quality levels (**Figure 2A**). The estimated compositions of the mock community varied widely at different quality scores. It is noteworthy that, when high quality trimming with a mean quality value (MQV) of 30 was employed, the genus *Filobasidiella* (dark green) accounted for almost 90% of the total community. The compositions for the genera *Acremonium* (blue), *Candida* (green), *Fusarium* (dark gray), and *Cryptococcus* (light blue) were more highly represented in the middle quality ranges, around MQV20, and decreased toward MQV30. The compositions of the genera *Saccharomyces* and *Nakaseomyces* were barely detected in this analysis, as its ITS1 regions consist of sequences that are longer than others (**Figure 2B**). Plotting MQV at each sequence position clearly revealed that the quality scores rapidly dropped to MQV20 at 50 bp, and decreased to MQV15 at 275 bp, the average length of all ITS1 regions (**Figure 2C**). When we performed hierarchical clustering analysis of the compositions at different quality scores (**Figure 2D**), the population compositions at MQV11 clustered with the original mock population. However, the original composition could not be reconstructed accurately when the MQV was set over 21. Mycobiota analysis using IonPGM depended largely on the length of the ITS1 regions. This shows that the quality of the reads must be carefully considered when using IonPGM. In our case, the quality trimming score of MQV 11 yielded the most accurate results.

### Mock Community Analysis by MiSeq Sequencing

**FIGURE 3:**
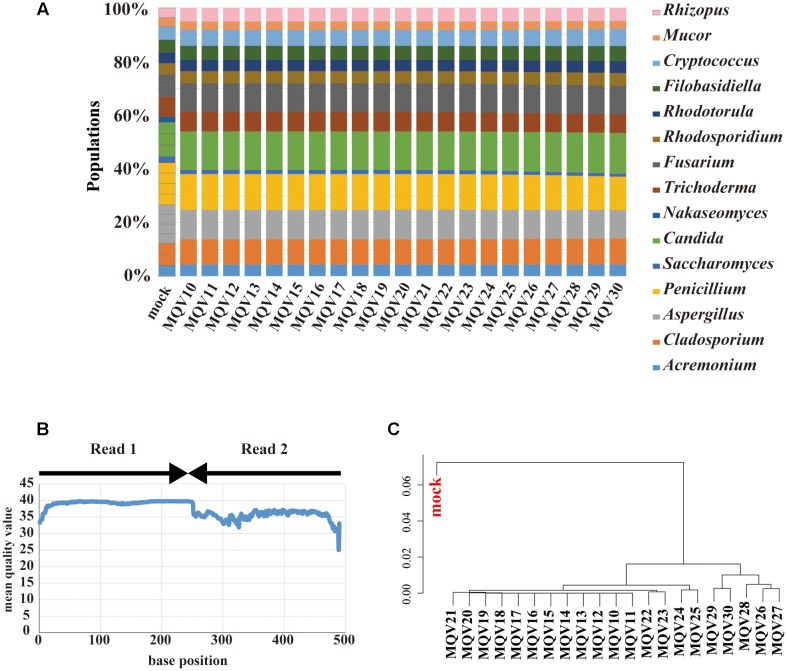
**Results of mycobiota analysis in the mock community using MiSeq sequencing.** **(A)** Relative abundance of major fungal genera in the mock community shown in a bar graph. Read-trimming was carried out using an ideal relative abundance of the mock community and MQV 10 – 30. Percentage read abundance of 15 genera are shown in **Supplementary Figure S4**. **(B)** MQV at each base position of the sequences merged with paired-end reads. **(C)** Genus-level hierarchical clustering analysis of the relative abundance of the major fungal genera. Results of analyses conducted using MQV ranging from 10 to 30 were analyzed along with ideal values.

MiSeq paired-end sequencing of 251 bp yielded 181,436 reads. These paired reads were merged, trimmed, and subjected to taxonomic assignment with various trimming conditions. Unlike the case of IonPGM, the estimated compositions at each MQV score were not influenced by the trimming quality (**Figure 3A**). However, the genus *Nakaseomyces* was not identified at any quality score. The composition of the genus *Saccharomyces* was estimated to be as low as approximately half of the original population. As stated above, the ITS1 regions of these specific genera have long sequences, around 500 bp. In particular, the ITS1 region of *Candida glabrata*, which was used as a representative of the genus *Nakaseomyces*, had an ITS1 length of 520 bp. This long ITS1 region could not be sequenced by the 251 bp paired-end sequencing method. Similar to the IonPGM data, the MQV plot of MiSeq also tended to decrease according to the length of the reads (**Figure 3B**). The overall MQVs of the read2 region are lower than those of the read1 region. However, because the overall MQVs were higher than 30, quality trimming had no impact. The hierarchical clustering analysis revealed that there are no large differences between MQVs. The cluster most closely resembling the mock population was MQV26-30 (**Figure 3C**). The analysis with MiSeq sequencing reconstructed nearly the entire population of the mock community, except the genus *Nakaseomyces* which was not identified.

### Mock Community Analysis by PacBio Sequencing

**FIGURE 4:**
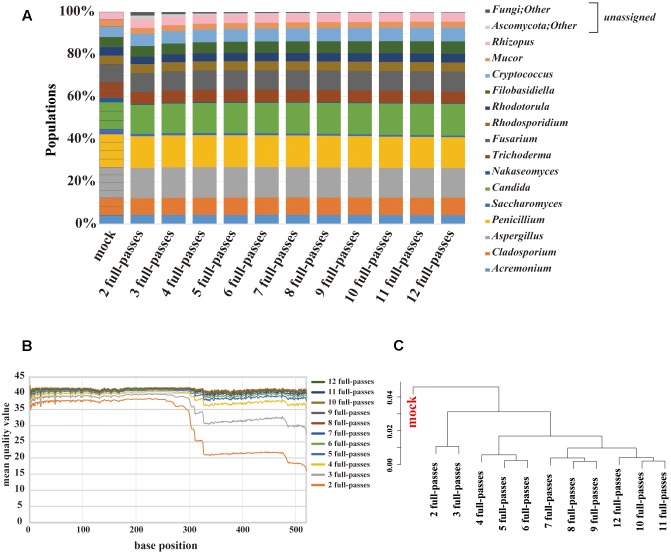
**Results of mycobiota analysis in the mock community using PacBio sequencing.** **(A)** Relative abundance of major fungal genera in the mock community shown in a bar graph. Consensus sequences were created with the ideal values for the mock community and between 2 and 12 full passes. Percentage read abundance of 15 genera are shown in **Supplementary Figure S4**. **(B)** MQV at each base position of the consensus sequences created with varying numbers of full passes **(C)** Genus-level hierarchical clustering analysis of the relative abundance of the major fungal genera. Results of analyses conducted with between 2 and 12 full passes were analyzed along with ideal values.

PacBio sequencing using one cell yielded 2,189,947 subreads. The MQV of these subreads was 9.5, which was extremely low. To increase sequence accuracy, circular consensus sequencing (CCS) was employed. As the consensus sequence was generated, the number of full passes used in the analysis ranged from 2 to 12. At two full-passes, 72,406 reads were obtained. At 12 full-passes, 40,934 reads were obtained. We evaluated the composition change according to the numbers of full-passes (**Figure 4A**). The PacBio sequencing reconstructed the composition of the original mock community, including the genera *Nakaseomyces* and *Saccharomyces.* Among the data associated with a small number of full-passes, sequences that could not be identified, such as those labeled as “fungi” or “other,” were confirmed to account for less than 10% of sequences, and were not present in data sets with a higher number of full-passes. The MQV plot of PacBio showed that MQVs of reads longer than 300 bp increased according to the number of full-passes (**Figure 4B**). At the 520 bp position, consensus sequences with four and eight full-passes achieved MQV30 and MQV40, respectively. The hierarchical clustering analysis revealed that there are no large differences related to the number of full-passes (**Figure 4C**). Therefore, the consensus sequence with eight full-passes, which could identify 98% or more of all 15 fungal genera, was considered to have been the optimal analytical parameters for PacBio sequencing, and those parameters were used for subsequent analysis of the gut mycobiota of healthy Japanese individuals.

### Gut Mycobiota in Healthy Japanese Individuals

**FIGURE 5:**
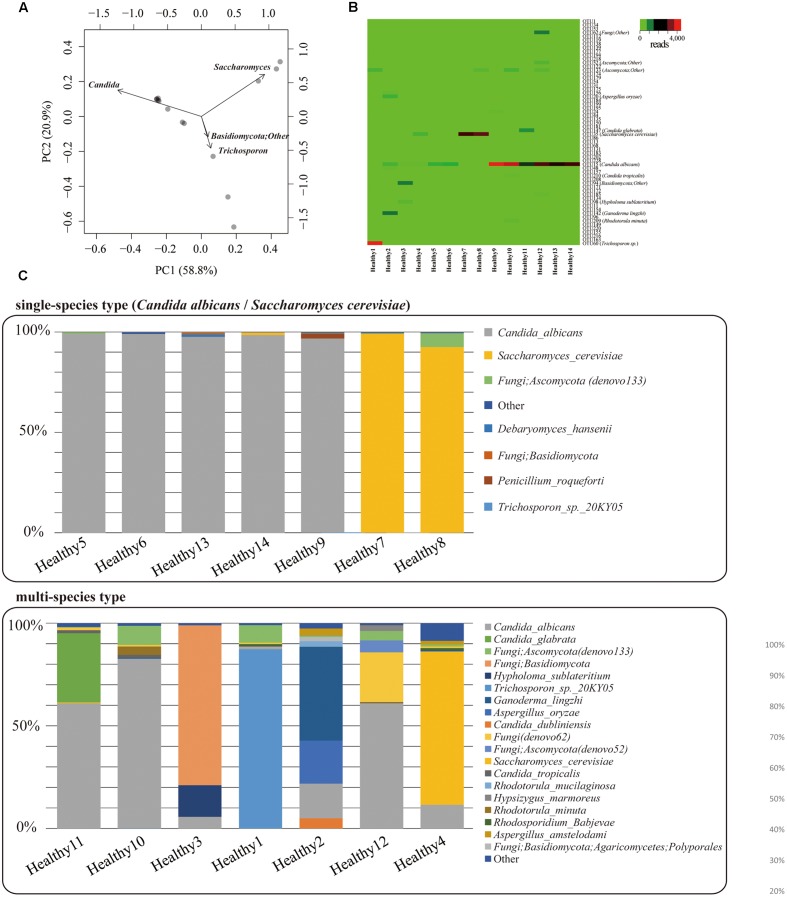
**Gut mycobiota in healthy Japanese individuals.** **(A)** Genus-level principal component analysis of mycobiota in the feces of 14 healthy Japanese subjects. Each individual subject is shown with a black dot. **(B)** Heat-map [operational taxonomic unit (OTU) level] of mycobiota found in the feces of the 14 healthy Japanese subjects. Number of reads for each OTU is shown in a color scale ranging from green to red (low to high). Name of each OTU is listed in **Supplementary Table S4**. **(C)** Bar charts of the mycobiota found in the feces of each of the Japanese subjects. The upper graph represents the “single species type” the bottom one represents the “multi-species type.”

Mycobiota analysis was performed on fecal samples from 14 healthy subjects. PacBio consensus sequences with eight full-passes yielded an average of 2,984 reads per sample. We performed the PCA on mycobiota at the genus level for 14 individuals (**Figure 5A**), and characterized two major mycobiota types, Genera *Candida*-dominant and *Saccharomyces* dominant type. A third group composed of other fungi was also identified. Relationships between mycobiota and gender or age differences were analyzed, but no apparent differences were found (**Supplementary Figure S2**). The heat map of fungal OTUs revealed a low diversity among the major fungi present in each individual (**Figure 5B**). The mycobiota of seven individuals was composed of either only a single species or was >90% of *C. albicans* or *S. cerevisiae* (**Figure 5C**). For the remaining seven individuals, the mycobiota were composed of multiple species, including *C. glabrata. C. dubliniensis. Ganoderma lingzhi. Aspergillus oryzae*, and unidentified fungal sequences. All fungi found in feces are listed in **Supplementary Table S6**. We determined that the mycobiota of healthy Japanese individuals is comprised of a simple assemblage consisting of only one or a few species.

## Discussion

### Comparison of IonPGM, MiSeq, and PacBio ITS1 Sequencing for Characterization of a Fungal Community

**FIGURE 6:**
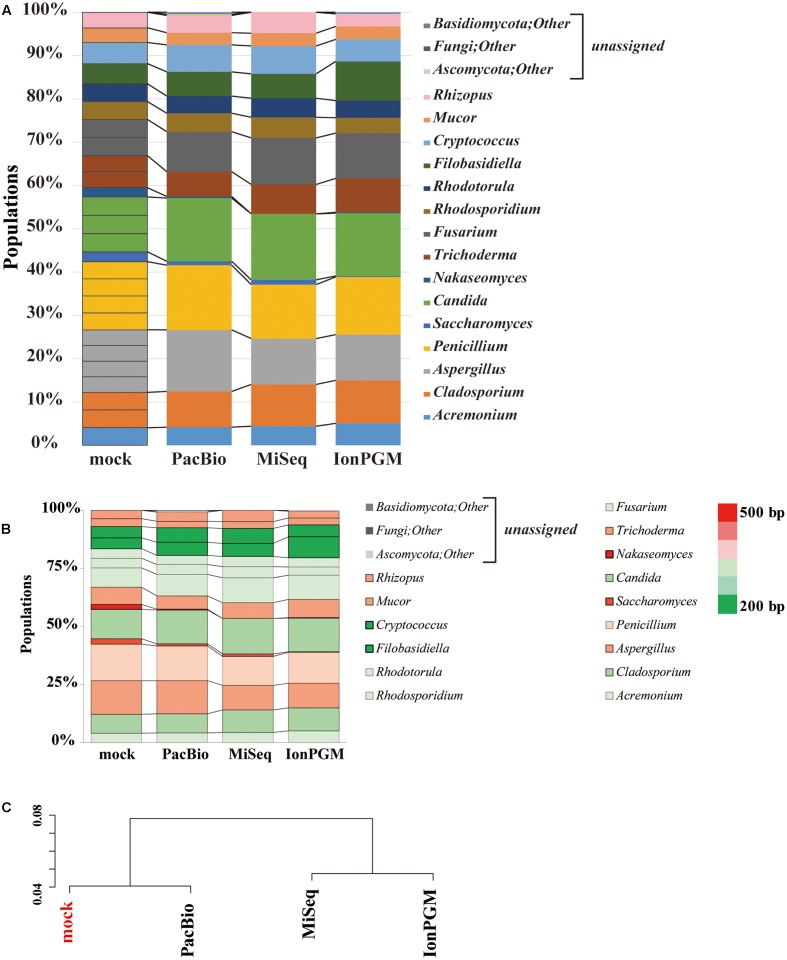
**Comparison of IonPGM, MiSeq, and PacBio sequencing performance for ITS1 fungal community.** **(A)** Relative abundance of major fungal genera in the mock community. The theoretical proportion for the mock community together with the optimal analysis of the results obtained by PacBio, MiSeq, and IonPGM sequencing are shown in a bar graph. Percentage read abundance of 15 genera are shown in **Supplementary Figure S4**. **(B)** Relative abundance of the major fungal genera found in the mock community are shown in different colors according to length of the ITS1 sequence. **(C)** Results of the hierarchical clustering of the composition of the major fungal genera derived from the results obtained by each sequencing method.

We evaluated the performance of three sequencing technologies, IonPGM, MiSeq, and PacBio, for an ITS1 deep sequencing analysis of a 26-species fungal community. **Figure 6A** summarizes the results obtained from surveys of each sequencing technology. For IonPGM sequencing, relative abundances of genera *Nakaseomyces* and *Saccharomyces* were evaluated to be less than 10% of the original mock while the genus *Filobasidiella* was estimated to be twice as much. For MiSeq sequencing, the genus *Nakaseomyces* was not identified, and the genus *Saccharomyces* was estimated at less than half of the original mock population. In addition, the abundances of genera *Aspergillus. Cryptococcus*, and *Rhizopus* deviated by approximately 30% from the original mock. For PacBio sequencing, the relative abundances of genera *Nakaseomyces* and *Saccharomyces* were underestimated, with results suggesting less than half of the concentration of the original mock population, while the relative abundances of the other genera were estimated accurately. While second-generation sequencing, such as IonPGM and MiSeq, tends to read shorter DNA sequences, PacBio has less of a length-dependent sequencing bias, however, the length dependence cannot be eliminated completely (Fichot and Norman, 2013).

We generated a heat map of the relative abundances for each sequencing technology according to the length of the ITS1 region of each fungal genus (**Figure 6B**). For all sequencing technologies, the longer ITS1 sequences, shown in red, were underestimated from the original mock sample, and the shorter ITS1, which are shown in green, were overestimated. The ITS1 regions of *C. glabrata* and *S. cerevisiae* are approximately 500 bp. Therefore, it is impossible to sequence them in their entirety using the current IonPGM sequencing kit, which allows for reading DNA sequences up to a maximum length of 400 bp. *C. glabrata* is a fungal species commonly present in humans (Underhill and Iliev, 2014) and *S. cerevisiae* is a very well-known species included in common food products. Therefore, these two species of fungi are highly likely to be present in the human mycobiome.

Despite the importance of the two major species, *C. glabrata* was not found with MiSeq or IonPGM sequencing. Pyrosequencing technology such as IonPGM sequencing and 454 sequencing (Roche) are currently the most widely used methods for the analysis of mycobiota. However, pyrosequencing results contain many homopolymer errors, and all services pertaining to 454 sequencing will be unavailable after 2016. In addition, IonPGM sequencing is fundamentally dependent on the sequence length. Even though the Hi-Q sequencing chemistry, which reduces insert-deletion error compared with previous version, was applied in this study, sequence quality decay was observed (**Supplementary Figure S3**). Compared with IonPGM, MiSeq was found to be more suitable for mycobiota analysis. These results were consistent with recently reported findings that compared MiSeq with IonPGM analysis (Tang et al., 2015).

Our study using PacBio revealed that all fungi from our mock community could be accurately assigned. The hierarchical clustering analysis of results from each sequencing analysis revealed that PacBio provided the most accurate estimation of the mock community population (**Figure 6C**). This was particularly true for some species of fungi with ITS1 regions as long as 800 bp. In the further studies aiming to assign all OTUs at the species level, expanding the fungal sequence database and conducting an analysis of the entire length of the ITS region by selecting different regions will be required. With our approach, the PacBio method was the only one that allowed for analysis of long sequence lengths with sufficient margins, covering the entire length of the ITS region. However, it must be noted that some fungi cannot be differentiated by the ITS region alone, which is a limitation of this analysis (Mello et al., 2011; Schoch et al., 2012; Tonge et al., 2014).

### Mycobiota Analysis of Healthy Japanese Individuals

Our study is the first report analyzing the mycobiota of the feces of Japanese individuals using deep ITS1 sequencing. We found that the mycobiota of most subjects were composed of only a few species, mainly consisting of genera *Candida* and *Saccharomyces*. These results were consistent with previous reports exploring the composition of fungi in human feces (Dollive et al., 2012; Hoffmann et al., 2013). In the genus *Candida*, we found the following species: *C. albicans. C. glabrata*, and *C. dubliniensis*, which have been reported as the most commonly found fungal species in humans (Underhill and Iliev, 2014; Romani et al., 2015). Our study revealed that while the two major genera in the mycobiota were *Candida* and *Saccharomyces*, some people were carriers of other populations of mycobiota. These mycobiomes were composed of identifiable fungi such as *Tricosporon* spp., *G. lingzhi. Hypsizygus sublateritium*, and *A. oryzae*, as well as a large number of sequences that could not be assigned to a known taxa.

Although the mycobiota of each individual participant did not show much diversity, the identification of those unknown minor fungi in the mycobiota would be the next targets for research on human commensal microorganisms. Further development of fungal genome databases will be essential for the analysis of various mycobiota and diseases associated with specific commensal microorganisms.

## Author Contributions

DM and SN designed the study, performed experiments and data analysis, interpreted the analyzed results, and coauthored the manuscript. KF and RT performed experiments and coauthored the manuscript. KG, YM, YF, TK, NG, TeY, MN, AK, and TH contributed valuable advice on the analyzed results. TaY, TI, and KT designed the study, coordinated research and helped to author the manuscript. All authors have read and approved the final manuscript.

## Conflict of Interest Statement

The authors declare that the research was conducted in the absence of any commercial or financial relationships that could be construed as a potential conflict of interest.

## Footnotes

1 https://bitbucket.org/daisukem/fungi/wiki/Home

2 http://sourceforge.net/projects/bbmap/

3 http://hannonlab.cshl.edu/fastx_toolkit/

4 http://sco.h-its.org/exelixis/web/software/pear/
