# Supplementary material for: Fungal ITS1 Deep-Sequencing Strategies to Reconstruct the Composition of a 26-Species Community and Evaluation of the Gut Mycobiota of Healthy Japanese Individuals
Source: Front Microbiol. 2017 Feb 15;8:238. doi: 10.3389/fmicb.2017.00238 (PMC5309391; doi:10.3389/fmicb.2017.00238)
Supplement: Supplementary file 1 [file Table_1.PDF]

Table S1. Characteristics of previous ITS databases and our database

Database Name	region	sequences[a]	Genera[a]	Species[a]	Average length[a]
**ntF-ITS1**	ITS1	13,943	1,218	6,525	257
**THF[b]**	ITS1-ITS2	2,341	317	1,718	232
**UNITE[c]**	ITS1-ITS2	25,716	2,323	11,655	227
**Findley[d]**	ITS1-ITS2	2,023	611	-[e]	270

[a] For THF, UNITE and Findley, we extacted ITS1 region using in the same manner as described in Methods

[b] THF version is 1.3

[c] UNITE version is 7.1. We used database from clustering at the 99% threshold level.

[d] The ITS database was downloaded from the Segre Lab at NHGRI (https://www.mothur.org/w/images/2/20/Findley_ITS_database.zip)

[e] There is no data for species name.
