# Supplementary material for: Fungal ITS1 Deep-Sequencing Strategies to Reconstruct the Composition of a 26-Species Community and Evaluation of the Gut Mycobiota of Healthy Japanese Individuals
Source: Front Microbiol. 2017 Feb 15;8:238. doi: 10.3389/fmicb.2017.00238 (PMC5309391; doi:10.3389/fmicb.2017.00238)
Supplement: Supplementary file 2 [file Table_2.PDF]

Table S2. Results of the clustering for each ITS1 sequence of the fungi in the mock community.

Clustering at 100 - 98% identity

denovo0	*Saccharomyces cerevisiae*	
denovo1	*Acremonium alternatum*	
denovo2	*Trichoderma viride*	*Trichoderma koningii*
denovo3	*Penicillium digitatum*	
denovo4	*Fusarium solani*	
denovo5	*Cryptococcus neoformans*	
denovo6	*Candida albicans*	
denovo7	*Fusarium oxysporum*	
denovo8	*Aspergillus flavus*	
denovo9	*Rhodosporidium babjevae*	
denovo10	*Candida dubliniensis*	
denovo11	*Aspergillus terreus*	
denovo12	*Mucor ramosissimus*	
denovo13	*Cladosporium cladosporioides*	
denovo14	*Penicillium chrysogenum*	
denovo15	*Penicillium citrinum*	
denovo16	*Aspergillus niger*	
denovo17	*Rhodotorula mucilaginosa*	
denovo18	*Penicillium oxalicum*	
denovo19	*Candida glabrata*	
denovo20	*Cladosporium herbarum*	
denovo21	*Cryptococcus aureus*	
denovo22	*Aspergillus fumigatus*	
denovo23	*Candida tropicalis*	
denovo24	*Rhizopus oryzae*	

Clustering at 97% identity

denovo0	*Saccharomyces cerevisiae*	
denovo1	*Acremonium alternatum*	
denovo2	*Trichoderma viride*	*Trichoderma koningii*

denovo3	*Penicillium digitatum*	*Penicillium chrysogenum*
denovo4	*Fusarium solani*	
denovo5	*Cryptococcus neoformans*	
denovo6	*Candida albicans*	
denovo7	*Fusarium oxysporum*	
denovo8	*Aspergillus flavus*	
denovo9	*Rhodosporidium babjevae*	
denovo10	*Rhodotorula mucilaginosa*	
denovo11	*Aspergillus terreus*	
denovo12	*Mucor ramosissimus*	
denovo13	*Cladosporium cladosporioides*	
denovo14	*Penicillium citrinum*	
denovo15	*Aspergillus niger*	
denovo16	*Candida dubliniensis*	
denovo17	*Penicillium oxalicum*	
denovo18	*Candida glabrata*	
denovo19	*Cladosporium herbarum*	
denovo20	*Cryptococcus aureus*	
denovo21	*Aspergillus fumigatus*	
denovo22	*Candida tropicalis*	
denovo23	*Rhizopus oryzae*	

Clustering at 96% identity

denovo0	*Saccharomyces cerevisiae*	
denovo1	*Acremonium alternatum*	
denovo2	*Trichoderma viride*	*Trichoderma koningii*
denovo3	*Penicillium digitatum*	*Penicillium chrysogenum*
denovo4	*Fusarium solani*	
denovo5	*Cryptococcus neoformans*	
denovo6	*Candida albicans*	
denovo7	*Fusarium oxysporum*	
denovo8	*Aspergillus flavus*	
denovo9	*Rhodosporidium babjevae*	

denovo10	*Rhodotorula mucilaginosa*	
denovo11	*Aspergillus terreus*	
denovo12	*Mucor ramosissimus*	
denovo13	*Penicillium citrinum*	
denovo14	*Aspergillus niger*	
denovo15	*Candida dubliniensis*	
denovo16	*Penicillium oxalicum*	
denovo17	*Candida glabrata*	
denovo18	*Cladosporium herbarum*	*Cladosporium cladosporioides*
denovo19	*Cryptococcus aureus*	
denovo20	*Aspergillus fumigatus*	
denovo21	*Candida tropicalis*	
denovo22	*Rhizopus oryzae*	

Clustering at 95% identity

denovo0	*Saccharomyces cerevisiae*	
denovo1	*Acremonium alternatum*	
denovo2	*Trichoderma viride*	*Trichoderma koningii*
denovo3	*Penicillium digitatum*	*Penicillium chrysogenum*
denovo4	*Fusarium solani*	
denovo5	*Cryptococcus neoformans*	
denovo6	*Candida albicans*	*Candida dubliniensis*
denovo7	*Fusarium oxysporum*	
denovo8	*Aspergillus flavus*	
denovo9	*Rhodosporidium babjevae*	
denovo10	*Rhodotorula mucilaginosa*	
denovo11	*Aspergillus terreus*	
denovo12	*Mucor ramosissimus*	
denovo13	*Penicillium citrinum*	
denovo14	*Aspergillus niger*	
denovo15	*Penicillium oxalicum*	
denovo16	*Candida glabrata*	
denovo17	*Cladosporium herbarum*	*Cladosporium cladosporioides*

denovo18	*Cryptococcus aureus*	
denovo19	*Aspergillus fumigatus*	
denovo20	*Candida tropicalis*	
denovo21	*Rhizopus oryzae*	

Clustering at 94 - 93% identity

denovo0	*Mucor ramosissimus*	
denovo1	*Cryptococcus neoformans*	
denovo2	*Aspergillus terreus*	
denovo3	*Candida albicans*	*Candida dubliniensis*
denovo4	*Aspergillus fumigatus*	
denovo5	*Aspergillus flavus*	
denovo6	*Fusarium oxysporum*	
denovo7	*Acremonium alternatum*	
denovo8	*Cladosporium herbarum*	*Cladosporium cladosporioides*
denovo9	*Penicillium oxalicum*	
denovo10	*Penicillium citrinum*	
denovo11	*Aspergillus niger*	
denovo12	*Trichoderma viride*	*Trichoderma koningii*
denovo13	*Saccharomyces cerevisiae*	
denovo14	*Rhodotorula mucilaginosa*	*Rhodosporidium babjevae*
denovo15	*Candida tropicalis*	
denovo16	*Cryptococcus aureus*	
denovo17	*Penicillium digitatum*	*Penicillium chrysogenum*
denovo18	*Fusarium solani*	
denovo19	*Candida glabrata*	
denovo20	*Rhizopus oryzae*	

Clustering at 92% identity

denovo0	*Mucor ramosissimus*	
denovo1	*Cryptococcus neoformans*	
denovo2	*Candida albicans*	*Candida dubliniensis*
denovo3	*Aspergillus fumigatus*	

denovo4	*Aspergillus flavus*			
denovo5	*Fusarium oxysporum*			
denovo6	*Acremonium alternatum*			
denovo7	*Cladosporium herbarum*	*Cladosporium cladosporioides*		
denovo8	*Penicillium oxalicum*			
denovo9	*Penicillium citrinum*			
denovo10	*Aspergillus niger*	*Aspergillus terreus*		
denovo11	*Trichoderma viride*	*Trichoderma koningii*		
denovo12	*Saccharomyces cerevisiae*			
denovo13	*Rhodotorula mucilaginosa*	*Rhodosporidium babjevae*		
denovo14	*Candida tropicalis*			
denovo15	*Cryptococcus aureus*			
denovo16	*Penicillium digitatum*	*Penicillium chrysogenum*		
denovo17	*Fusarium solani*			
denovo18	*Candida glabrata*			
denovo19	*Rhizopus oryzae*			

Clustering at 91% identity

denovo0	*Mucor ramosissimus*			
denovo1	*Cryptococcus neoformans*			
denovo2	*Candida albicans*	*Candida dubliniensis*		
denovo3	*Saccharomyces cerevisiae*			
denovo4	*Aspergillus flavus*			
denovo5	*Fusarium oxysporum*			
denovo6	*Acremonium alternatum*			
denovo7	*Cladosporium herbarum*	*Cladosporium cladosporioides*		
denovo8	*Cryptococcus aureus*			
denovo9	*Penicillium citrinum*			
denovo10	*Aspergillus niger*	*Aspergillus terreus*		
denovo11	*Trichoderma viride*	*Trichoderma koningii*		
denovo12	*Rhodotorula mucilaginosa*	*Rhodosporidium babjevae*		
denovo13	*Candida tropicalis*			
denovo14	*Penicillium digitatum*	*Penicillium chrysogenum*	*Penicillium oxalicum*	*Aspergillus fumigatus*

denovo15	*Fusarium solani*				
denovo16	*Candida glabrata*				
denovo17	*Rhizopus oryzae*				

Clustering at 90% identity

denovo0	*Mucor ramosissimus*				
denovo1	*Cryptococcus neoformans*				
denovo2	*Candida albicans*	*Candida dubliniensis*			
denovo3	*Saccharomyces cerevisiae*				
denovo4	*Aspergillus flavus*				
denovo5	*Fusarium oxysporum*				
denovo6	*Acremonium alternatum*				
denovo7	*Cladosporium herbarum*	*Cladosporium cladosporioides*			
denovo8	*Cryptococcus aureus*				
denovo9	*Aspergillus niger*	*Aspergillus terreus*			
denovo10	*Trichoderma viride*	*Trichoderma koningii*			
denovo11	*Rhodotorula mucilaginosa*	*Rhodosporidium babjevae*			
denovo12	*Candida tropicalis*				
denovo13	*Penicillium digitatum*	*Penicillium chrysogenum*	*Penicillium citrinum*	*Penicillium oxalicum*	*Aspergillus fumigatus*
denovo14	*Fusarium solani*				
denovo15	*Candida glabrata*				
denovo16	*Rhizopus oryzae*				
