# Supplementary material for: Fungal ITS1 Deep-Sequencing Strategies to Reconstruct the Composition of a 26-Species Community and Evaluation of the Gut Mycobiota of Healthy Japanese Individuals
Source: Front Microbiol. 2017 Feb 15;8:238. doi: 10.3389/fmicb.2017.00238 (PMC5309391; doi:10.3389/fmicb.2017.00238)
Supplement: Supplementary file 3 [file Table_3.PDF]

Table S3. Results of the taxonomic assignment for each ITS1 sequence of the fungi in the mock community.

Fungi	species	genus	assigned Taxonomy		
			RDP	blastn	UCLUST
1	*Acremonium alternatum*	*Acremonium*	*Acremonium*	*Sarocladium*	*Acremonium*
2	*Aspergillus flavus*	*Aspergillus*	*Aspergillus*	*Aspergillus*	*Aspergillus*
3	*Aspergillus fumigatus*	*Aspergillus*	*Aspergillus*	*Aspergillus*	*Aspergillus*
4	*Aspergillus niger*	*Aspergillus*	*Aspergillus*	*Aspergillus*	*Aspergillus*
5	*Aspergillus terreus*	*Aspergillus*	*Aspergillus*	*Aspergillus*	unassigned
6	*Candida albicans*	*Candida*	*Candida*	*Candida*	*Candida*
7	*Candida dubliniensis*	*Candida*	*Candida*	*Candida*	*Candida*
8	*Candida tropicalis*	*Candida*	*Candida*	*Candida*	*Candida*
9	*Cladosporium cladosporioides*	*Cladosporium*	*Cladosporium*	*Cladosporium*	*Cladosporium*
10	*Cladosporium herbarum*	*Cladosporium*	*Cladosporium*	*Cladosporium*	*Cladosporium*
11	*Cryptococcus aureus*	*Cryptococcus*	*Cryptococcus*	*Puccinia*	unassigned
12	*Cryptococcus neoformans*	*Filobasidiella*	*Filobasidiella*	*Filobasidiella*	*Filobasidiella*
13	*Fusarium oxysporum*	*Fusarium*	*Fusarium*	*Fusarium*	*Fusarium*
14	*Fusarium solani*	*Fusarium*	*Fusarium*	*Fusarium*	*Fusarium*
15	*Mucor ramosissimus*	*Mucor*	*Mucor*	*Mucor*	*Mucor*
16	*Candida glabrata*	*Nakaseomyces*	*Nakaseomyces*	*Nakaseomyces*	unassigned
17	*Penicillium chrysogenum*	*Penicillium*	*Penicillium*	*Penicillium*	*Penicillium*
18	*Penicillium citrinum*	*Penicillium*	*Penicillium*	*Penicillium*	unassigned
19	*Penicillium digitatum*	*Penicillium*	*Penicillium*	*Penicillium*	*Penicillium*
20	*Penicillium oxalicum*	*Penicillium*	*Penicillium*	*Penicillium*	*Penicillium*
21	*Rhizopus oryzae*	*Rhizopus*	*Rhizopus*	*Rhizopus*	*Rhizopus*
22	*Rhodosporidium babjevae*	*Rhodosporidium*	*Rhodosporidium*	*Rhodotorula*	unassigned
23	*Rhodotorula mucilaginosa*	*Rhodotorula*	*Rhodotorula*	*Rhodotorula*	*Rhodotorula*
24	*Saccharomyces cerevisiae*	*Saccharomyces*	*Saccharomyces*	*Saccharomyces*	*Saccharomyces*
25	*Trichoderma viride*	*Trichoderma*	*Trichoderma*	*Trichoderma*	*Trichoderma*
26	*Trichoderma koningii*	*Trichoderma*	*Trichoderma*	*Trichoderma*	*Trichoderma*
