# Supplementary material for: Fungal ITS1 Deep-Sequencing Strategies to Reconstruct the Composition of a 26-Species Community and Evaluation of the Gut Mycobiota of Healthy Japanese Individuals
Source: Front Microbiol. 2017 Feb 15;8:238. doi: 10.3389/fmicb.2017.00238 (PMC5309391; doi:10.3389/fmicb.2017.00238)
Supplement: Supplementary file 4 [file Table_4.PDF]

Table S4. Results of the clustering and taxonomic assignment for fungal sequences

**OTUs**	**Classified species**	**Assigned Taxonomy (genus level)**
OTU0	*Saccharomyces cerevisiae*	*Saccharomyces*
OTU1	*Acremonium alternatum*	*Acremonium*
OTU2	*Trichoderma viride*	*Trichoderma*
	Trichoderma koningii	
OTU3	*Penicillium digitatum*	*Penicillium*
	Penicillium chrysogenum	
OTU4	*Fusarium solani*	*Fusarium*
OTU5	*Cryptococcus neoformans*	*Filobasidiella*
OTU6	*Candida albicans*	*Candida*
	Candida dubliniensis	
OTU7	*Fusarium oxysporum*	*Fusarium*
OTU8	*Aspergillus flavus*	*Aspergillus*
OTU9	*Rhodosporidium babjevae*	*Rhodosporidium*
OTU10	*Rhodotorula mucilaginosa*	*Rhodotorula*
OTU11	*Aspergillus terreus*	*Aspergillus*
OTU12	*Mucor ramosissimus*	*Mucor*
OTU13	*Penicillium citrinum*	*Penicillium*
OTU14	*Aspergillus niger*	*Aspergillus*
OTU15	*Penicillium oxalicum*	*Penicillium*
OTU16	*Candida glabrata*	*Nakaseomyces*
OTU17	*Cladosporium herbarum*	*Cladosporium*
	Cladosporium cladosporioides	
OTU18	*Cryptococcus aureus*	*Cryptococcus aureus*
OTU19	*Aspergillus fumigatus*	*Aspergillus*
OTU20	*Candida tropicalis*	*Candida*
OTU21	*Rhizopus oryzae*	*Rhizopus*
