# Supplementary material for: Fungal ITS1 Deep-Sequencing Strategies to Reconstruct the Composition of a 26-Species Community and Evaluation of the Gut Mycobiota of Healthy Japanese Individuals
Source: Front Microbiol. 2017 Feb 15;8:238. doi: 10.3389/fmicb.2017.00238 (PMC5309391; doi:10.3389/fmicb.2017.00238)
Supplement: Supplementary file 5 [file Table_5.PDF]

Table S5. Results of the taxonomic assignment for fungal sequences using different database.

Fungi	species	genus	ntF-ITS1	UNITE[a]	THF[a]	Findley[a]
1	*Acremonium alternatum*	*Acremonium*	*Acremonium*	*Acremonium*	*Acremonium*	*Acremonium*
2	*Aspergillus flavus*	*Aspergillus*	*Aspergillus*	*Aspergillus*	*Aspergillus*	*unclassified_Trichocomaceae_genus*
3	*Aspergillus fumigatus*	*Aspergillus*	*Aspergillus*	*Trichocomaceae;unidentified*	*Aspergillus*	*Neosartorya;Other*
4	*Aspergillus niger*	*Aspergillus*	*Aspergillus*	*Aspergillus*	*Aspergillus*	*Aspergillus*
5	*Aspergillus terreus*	*Aspergillus*	*Aspergillus*	*Aspergillus*	*Aspergillus*	*Aspergillus*
6	*Candida albicans*	*Candida*	*Candida*	*Candida*	*Candida*	*Candida*
7	*Candida dubliniensis*	*Candida*	*Candida*	*Candida*	*Candida*	*Candida*
8	*Candida tropicalis*	*Candida*	*Candida*	*Candida*	*Candida*	*Candida*
9	*Cladosporium cladosporioides*	*Cladosporium*	*Cladosporium*	*Ascomycota;unidentified*	*Cladosporium*	*Cladosporium*
10	*Cladosporium herbarum*	*Cladosporium*	*Cladosporium*	*Mycosphaerella*	*Cladosporium*	*unclassified_Capnodiales_genus*
11	*Cryptococcus aureus*	*Cryptococcus*	*Cryptococcus*	*Cryptococcus*	*Cryptococcus*	*unclassified_Tremellales_genus*
12	*Cryptococcus neoformans*	*Filobasidiella*	*Filobasidiella*	*Cryptococcus*	*Filobasidiella*	*Filobasidiella*
13	*Fusarium oxysporum*	*Fusarium*	*Fusarium*	*Fusarium*	*Fusarium*	*Fusarium*
14	*Fusarium solani*	*Fusarium*	*Fusarium*	*Fusarium*	*Fusarium*	*Fusarium*
15	*Mucor ramosissimus*	*Mucor*	*Mucor*	*Mucor*	*Mucor*	*Mucor*
16	*Candida glabrata*	*Nakaseomyces*	*Nakaseomyces*	*Candida*	*Nakaseomyces*	*unclassified_Saccharomycetaceae_genus*
17	*Penicillium chrysogenum*	*Penicillium*	*Penicillium*	*Penicillium*	*Penicillium*	*Penicillium*
18	*Penicillium citrinum*	*Penicillium*	*Penicillium*	*Penicillium*	*Penicillium*	*Penicillium*
19	*Penicillium digitatum*	*Penicillium*	*Penicillium*	*Penicillium*	*Penicillium*	*Penicillium*
20	*Penicillium oxalicum*	*Penicillium*	*Penicillium*	*Penicillium*	*Penicillium*	*Penicillium*
21	*Rhizopus oryzae*	*Rhizopus*	*Rhizopus*	*Rhizopus*	*Rhizopus*	*Rhizopus*
22	*Rhodosporidium babjevae*	*Rhodosporidium*	*Rhodosporidium*	*Basidiomycota;unidentified*	*Rhodosporidium*	*Rhodotorula*
23	*Rhodotorula mucilaginosa*	*Rhodotorula*	*Rhodotorula*	*Rhodotorula*	*Rhodotorula*	*Rhodotorula*
24	*Saccharomyces cerevisiae*	*Saccharomyces*	*Saccharomyces*	*Saccharomyces*	*Saccharomyces*	*Saccharomyces*
25	*Trichoderma viride*	*Trichoderma*	*Trichoderma*	*Trichoderma*	*Trichoderma*	*Hypocrea* [b]
26	*Trichoderma koningii*	*Trichoderma*	*Trichoderma*	*Trichoderma*	*Trichoderma*	*Hypocrea* [b]

[a] For THF, UNITE and Findley, we extacted ITS1 region using in the same manner as described in Methods

[b] Hypocrea is the anamorphas of Trichoderma
