# Supplementary material for: Fungal ITS1 Deep-Sequencing Strategies to Reconstruct the Composition of a 26-Species Community and Evaluation of the Gut Mycobiota of Healthy Japanese Individuals
Source: Front Microbiol. 2017 Feb 15;8:238. doi: 10.3389/fmicb.2017.00238 (PMC5309391; doi:10.3389/fmicb.2017.00238)
Supplement: Supplementary file 6 [file Table_6.PDF]

Table S6. Results of the taxonomic assignment of each OTU found in healthy Japanese individuals.

#OTU ID	Assigned Taxonomy
denovo60	Fungi;Basidiomycota;Tremellomycetes;Tremellales;Tremellales,Other;Trichosporon;Trichosporon_sp._20KY05
denovo117	Fungi;Ascomycota;Saccharomycetes;Saccharomycetales;Saccharomycetales,Other;Candida;Candida_albicans
denovo35	Fungi;Ascomycota;Saccharomycetes;Saccharomycetales;Saccharomycetaceae;Saccharomyces;Saccharomyces_cerevisiae
denovo20	Fungi;Ascomycota;Eurotiomycetes;Eurotiales;Aspergillaceae;Aspergillus;Aspergillus_oryzae
denovo62	Fungi
denovo52	Fungi;Ascomycota
denovo133	Fungi;Ascomycota
denovo44	Fungi;Ascomycota;Saccharomycetes;Saccharomycetales;Debaryomycetaceae;Debaryomyces;Debaryomyces_hansenii
denovo171	Fungi;Basidiomycota
denovo185	Fungi;Basidiomycota;Agaricomycetes;Agaricales;Lyophyllaceae;Hypsizygus;Hypsizygus_marmoreus
denovo156	Fungi;Ascomycota;Eurotiomycetes;Eurotiales;Aspergillaceae;Aspergillus;Aspergillus_amstelodami
denovo1	Fungi
denovo34	Fungi
denovo53	Fungi
denovo112	Fungi
denovo119	Fungi
denovo138	Fungi
denovo139	Fungi
denovo177	Fungi
denovo194	Fungi
denovo218	Fungi
denovo122	Fungi;Ascomycota
denovo174	Fungi;Ascomycota
denovo179	Fungi;Ascomycota;Ascomycota,Other;Ascomycota,Other,Other;Ascomycota,Other,Other,Other;Acremonium;Acremonium_sp._P42E5
denovo54	Fungi;Ascomycota;Dothideomycetes;Capnodiales;Davidiellaceae;Cladosporium;Cladosporium_sp._TMS-2011
denovo31	Fungi;Ascomycota;Dothideomycetes;Pleosporales;Didymellaceae;Atradidymella;Atradidymella_muscivora
denovo175	Fungi;Ascomycota;Eurotiomycetes;Chaetothyriales;Herpotrichiellaceae;Exophiala;Exophiala_equina
denovo184	Fungi;Ascomycota;Eurotiomycetes;Eurotiales;Aspergillaceae;Penicillium;Penicillium_citrinum
denovo199	Fungi;Ascomycota;Eurotiomycetes;Eurotiales;Aspergillaceae;Penicillium;Penicillium_digitatum
denovo195	Fungi;Ascomycota;Eurotiomycetes;Eurotiales;Aspergillaceae;Penicillium;Penicillium_oxalicum
denovo24	Fungi;Ascomycota;Eurotiomycetes;Eurotiales;Aspergillaceae;Penicillium;Penicillium_roqueforti

denovo135	Fungi;Ascomycota;Saccharomycetes;Saccharomycetales;Dipodascaceae
denovo150	Fungi;Ascomycota;Saccharomycetes;Saccharomycetales;Dipodascaceae;Galactomyces;Galactomyces_sp._BPY-54
denovo181	Fungi;Ascomycota;Saccharomycetes;Saccharomycetales;Dipodascaceae;Galactomyces;Galactomyces_sp._E234
denovo147	Fungi;Ascomycota;Saccharomycetes;Saccharomycetales;Saccharomycetaceae;Nakaseomyces;Candida_glabrata
denovo86	Fungi;Ascomycota;Saccharomycetes;Saccharomycetales;Saccharomycetaceae;Zygosaccharomyces;Zygosaccharomyces_rouxii
denovo13	Fungi;Ascomycota;Saccharomycetes;Saccharomycetales;Saccharomycetales,Other;Candida
denovo68	Fungi;Ascomycota;Saccharomycetes;Saccharomycetales;Saccharomycetales,Other;Candida
denovo131	Fungi;Ascomycota;Saccharomycetes;Saccharomycetales;Saccharomycetales,Other;Candida
denovo183	Fungi;Ascomycota;Saccharomycetes;Saccharomycetales;Saccharomycetales,Other;Candida
denovo202	Fungi;Ascomycota;Saccharomycetes;Saccharomycetales;Saccharomycetales,Other;Candida
denovo228	Fungi;Ascomycota;Saccharomycetes;Saccharomycetales;Saccharomycetales,Other;Candida
denovo48	Fungi;Ascomycota;Saccharomycetes;Saccharomycetales;Saccharomycetales,Other;Candida;Candida_dubliniensis
denovo157	Fungi;Ascomycota;Saccharomycetes;Saccharomycetales;Saccharomycetales,Other;Candida;Candida_sake
denovo210	Fungi;Ascomycota;Saccharomycetes;Saccharomycetales;Saccharomycetales,Other;Candida;Candida_tropicalis
denovo208	Fungi;Ascomycota;Sordariomycetes;Hypocreales;Nectriaceae;Fusarium;Fusarium_culmorum
denovo94	Fungi;Basidiomycota
denovo172	Fungi;Basidiomycota
denovo134	Fungi;Basidiomycota;Agaricomycetes;Agaricales;Pleurotaceae;Pleurotus;Pleurotus_eryngii
denovo98	Fungi;Basidiomycota;Agaricomycetes;Agaricales;Strophariaceae;Hypholoma;Hypholoma_sublateritium
denovo11	Fungi;Basidiomycota;Agaricomycetes;Auriculariales;Auriculariaceae;Auricularia;Auricularia_auricula-judae
denovo154	Fungi;Basidiomycota;Agaricomycetes;Polyporales
denovo142	Fungi;Basidiomycota;Agaricomycetes;Polyporales;Ganodermataceae;Ganoderma;Ganoderma_lingzhi
denovo96	Fungi;Basidiomycota;Agaricomycetes;Russulales;Hericiaceae;Hericium;Hericium_coralloides
denovo209	Fungi;Basidiomycota;Basidiomycota,Other;Erythrobasidiales;Erythrobasidiales,Other;Rhodotorula;Rhodotorula_minuta
denovo149	Fungi;Basidiomycota;Basidiomycota,Other;Sporidiobolales;Sporidiobolales,Other;Rhodosporidium;Rhodosporidium_Babjevae
denovo220	Fungi;Basidiomycota;Basidiomycota,Other;Sporidiobolales;Sporidiobolales,Other;Rhodotorula;Rhodotorula_mucilaginosa
denovo155	Fungi;Basidiomycota;Tremellomycetes;Filobasidiales;Filobasidiales,Other;Cryptococcus;Cryptococcus_albidus
denovo216	Fungi;Basidiomycota;Tremellomycetes;Filobasidiales;Filobasidiales,Other;Cryptococcus;Cryptococcus_sp._FM-D1
denovo163	Fungi;Basidiomycota;Tremellomycetes;Tremellales;Tremellales,Other;Trichosporon;Trichosporon_faecale
