# Supplementary material for: Fungal ITS1 Deep-Sequencing Strategies to Reconstruct the Composition of a 26-Species Community and Evaluation of the Gut Mycobiota of Healthy Japanese Individuals
Source: Front Microbiol. 2017 Feb 15;8:238. doi: 10.3389/fmicb.2017.00238 (PMC5309391; doi:10.3389/fmicb.2017.00238)
Supplement: Supplementary file 7 [file Image_1.PDF]

(A) THF

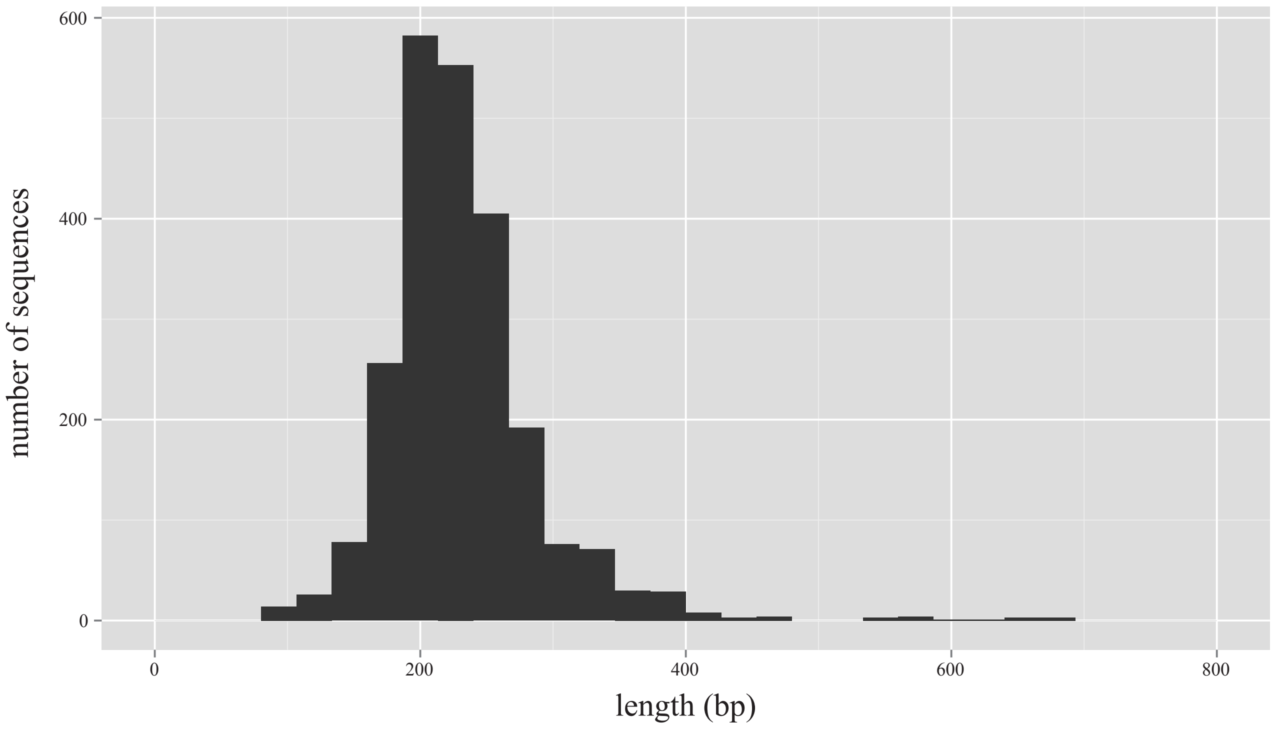

(B) UNITE

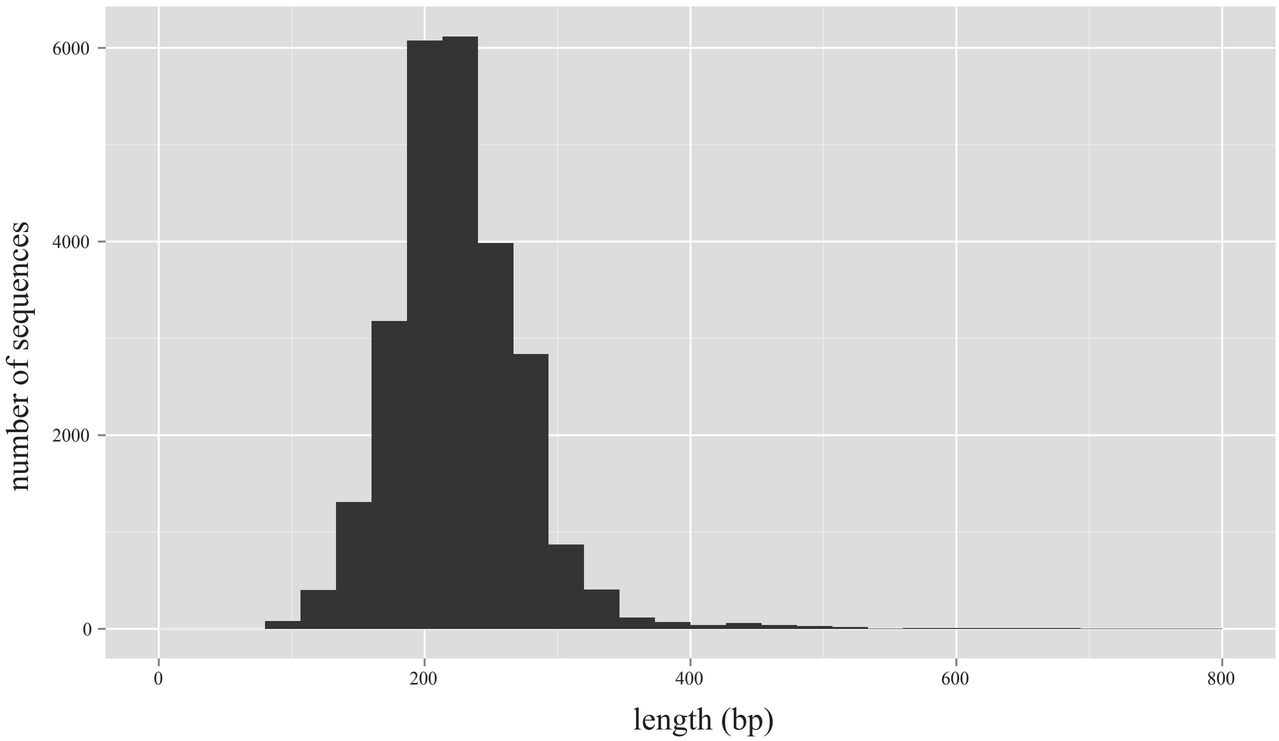

Fig. S1. The length distribution of ITS1 for each sequences in THF and UNITE

A histogram showing the variety in length distribution of fungal ITS1 region sequences. The horizontal axis represents the length of ITS1 sequences. The vertical axis represents the number of sequences of each length. (A) THF, (B) UNITE.
