# Supplementary material for: Fungal ITS1 Deep-Sequencing Strategies to Reconstruct the Composition of a 26-Species Community and Evaluation of the Gut Mycobiota of Healthy Japanese Individuals
Source: Front Microbiol. 2017 Feb 15;8:238. doi: 10.3389/fmicb.2017.00238 (PMC5309391; doi:10.3389/fmicb.2017.00238)
Supplement: Supplementary file 8 [file Image_2.PDF]

A

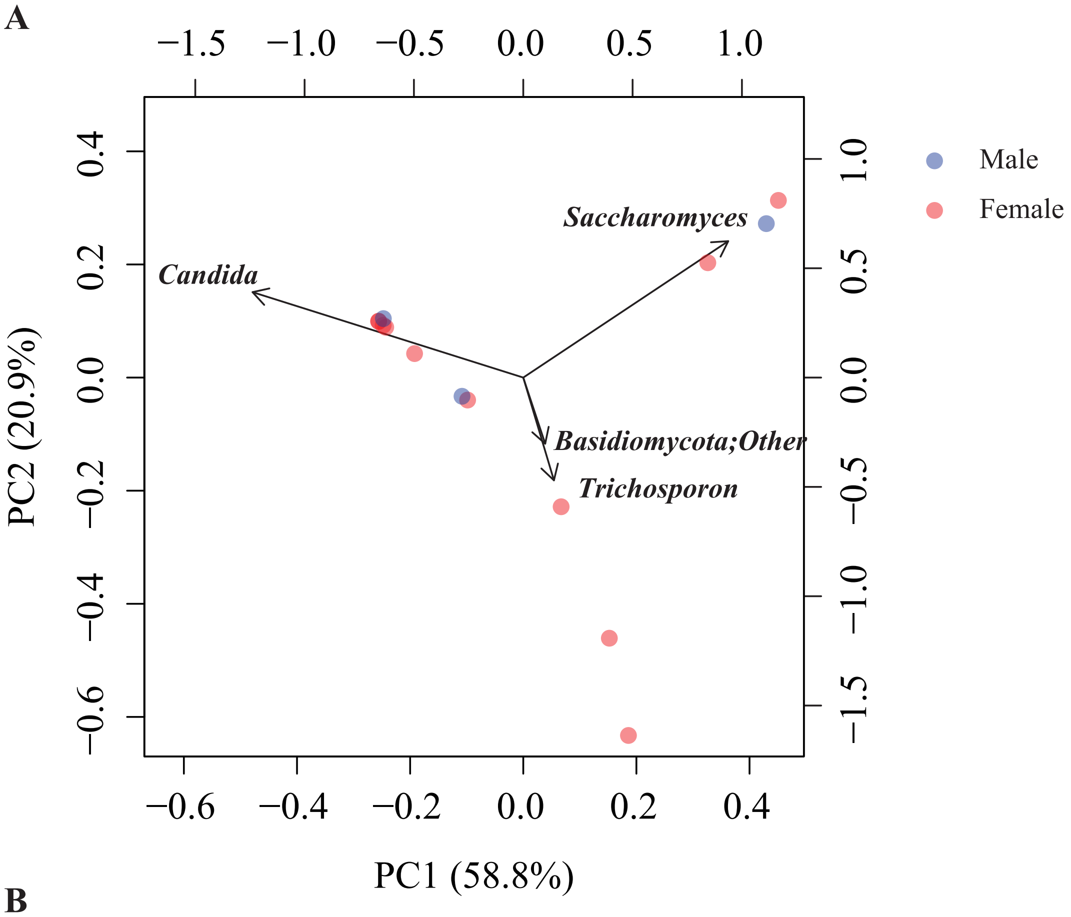

B

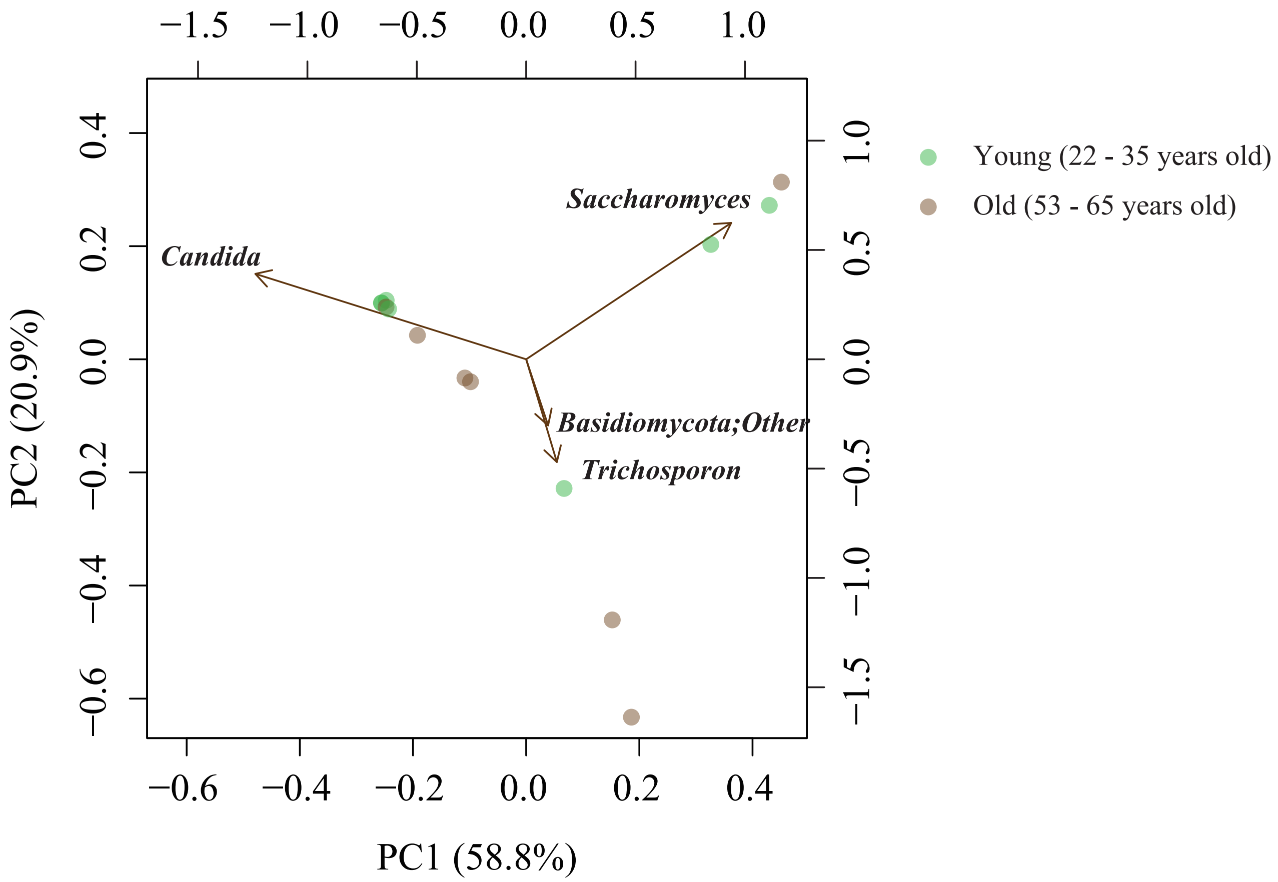

Fig. S2. Genus-level principal component analysis of the mycobiota of healthy Japanese individuals.
(A) Male and female subjects are shown separately in different colors: males are shown in purple, and females are shown in orange. (B) Young and old subjects are shown separately in different colors: young subjects are shown in green, and old subjects are shown in brown. "Young" included subjects aged between 22 and 35 years, and "old" included subjects aged between 53 and 65 years.
