# Supplementary material for: Fungal ITS1 Deep-Sequencing Strategies to Reconstruct the Composition of a 26-Species Community and Evaluation of the Gut Mycobiota of Healthy Japanese Individuals
Source: Front Microbiol. 2017 Feb 15;8:238. doi: 10.3389/fmicb.2017.00238 (PMC5309391; doi:10.3389/fmicb.2017.00238)
Supplement: Supplementary file 9 [file Image_3.PDF]

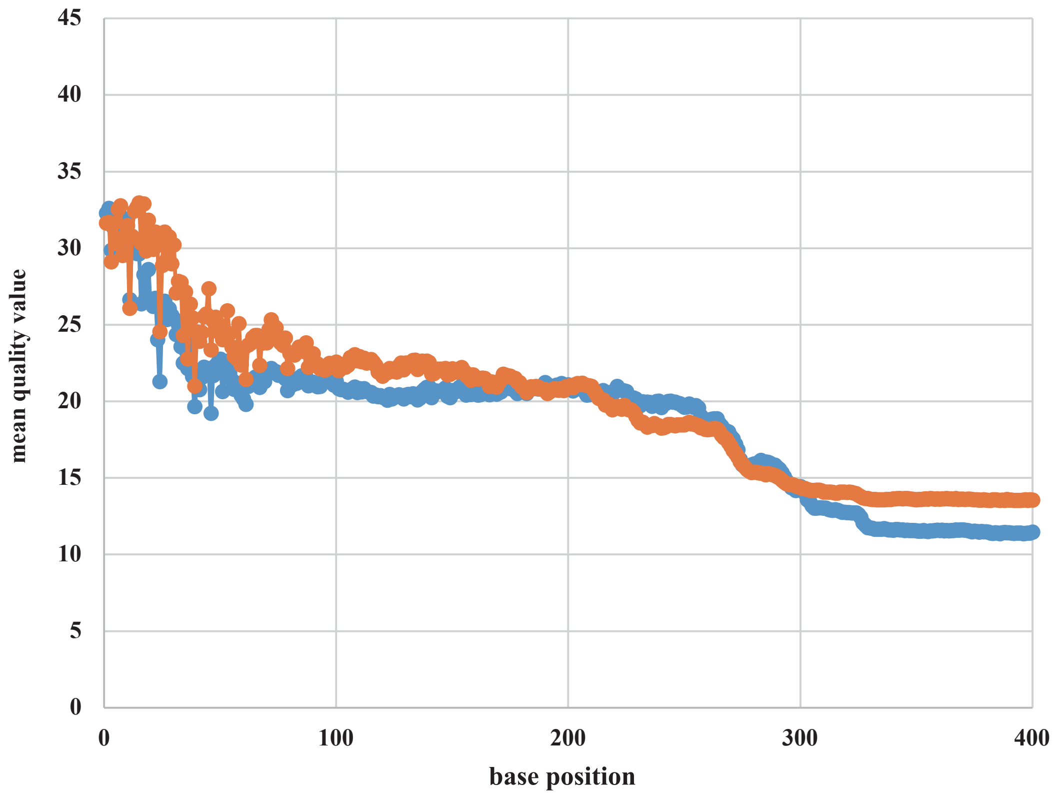

Fig. S3. Mean read quality of each base position for IonPGM.
MQV at each base position of the sequences obtained from sequencing with the Ion PGM Sequencing Hi-Q kit (blue) and the Ion PGM Sequencing 400 kit (orange).
