# Supplementary material for: Fungal ITS1 Deep-Sequencing Strategies to Reconstruct the Composition of a 26-Species Community and Evaluation of the Gut Mycobiota of Healthy Japanese Individuals
Source: Front Microbiol. 2017 Feb 15;8:238. doi: 10.3389/fmicb.2017.00238 (PMC5309391; doi:10.3389/fmicb.2017.00238)
Supplement: Supplementary file 10 [file Image_4.PDF]

# A

| Taxon | mock | MQV10 | MQV11 | MQV12 | MQV13 | MQV14 | MQV15 | MQV16 | MQV17 | MQV18 | MQV19 | MQV20 | MQV21 | MQV22 | MQV23 | MQV24 | MQV25 | MQV26 | MQV27 | MQV28 | MQV29 | MQV30 |
|---|---|---|---|---|---|---|---|---|---|---|---|---|---|---|---|---|---|---|---|---|---|---|
| Acremonium | 4.1% | 3.3% | 4.1% | 4.7% | 4.9% | 5.0% | 5.2% | 5.5% | 5.8% | 6.2% | 6.7% | 7.2% | 7.8% | 8.6% | 9.6% | 10.6% | 12.4% | 14.5% | 13.6% | 8.6% | 3.6% | 1.3% |
| Cladosporium | 8.2% | 6.8% | 8.4% | 9.6% | 10.0% | 9.9% | 9.9% | 10.0% | 10.4% | 10.8% | 10.9% | 10.5% | 9.9% | 9.1% | 8.0% | 6.6% | 5.1% | 3.9% | 3.1% | 2.7% | 1.7% | 0.4% |
| Aspergillus | 14.5% | 8.7% | 10.7% | 11.3% | 10.7% | 10.5% | 10.2% | 9.8% | 9.3% | 8.4% | 7.2% | 5.7% | 4.5% | 3.4% | 2.5% | 1.8% | 1.3% | 1.0% | 0.9% | 1.0% | 1.8% | 3.6% |
| Penicillium | 15.7% | 9.1% | 11.3% | 12.7% | 13.1% | 13.3% | 13.5% | 13.3% | 12.8% | 12.1% | 11.5% | 10.3% | 8.1% | 5.4% | 3.2% | 2.0% | 1.1% | 0.5% | 0.3% | 0.3% | 0.3% | 0.4% |
| Saccharomyces | 2.3% | 0.2% | 0.3% | 0.2% | 0.2% | 0.2% | 0.2% | 0.1% | 0.0% | 0.0% | 0.0% | 0.0% | 0.0% | 0.0% | 0.0% | 0.0% | 0.0% | 0.0% | 0.0% | 0.0% | 0.0% | 0.0% |
| Candida | 12.6% | 9.8% | 12.1% | 13.6% | 14.1% | 14.4% | 14.8% | 15.1% | 15.5% | 16.0% | 16.5% | 17.3% | 18.0% | 18.4% | 18.1% | 17.6% | 16.1% | 13.0% | 9.5% | 6.2% | 3.8% | 2.1% |
| Nakaseomyces | 2.1% | 0.3% | 0.4% | 0.4% | 0.3% | 0.2% | 0.1% | 0.1% | 0.0% | 0.0% | 0.0% | 0.0% | 0.0% | 0.0% | 0.0% | 0.0% | 0.0% | 0.0% | 0.0% | 0.0% | 0.0% | 0.0% |
| Trichoderma | 7.4% | 4.8% | 6.0% | 7.1% | 7.5% | 7.8% | 7.8% | 7.9% | 7.7% | 7.5% | 7.5% | 7.4% | 7.1% | 6.3% | 5.1% | 3.8% | 2.9% | 2.1% | 1.3% | 0.4% | 0.2% | 0.0% |
| Fusarium | 8.4% | 7.2% | 8.8% | 9.8% | 10.1% | 10.4% | 10.7% | 11.2% | 11.8% | 12.4% | 12.8% | 13.3% | 14.0% | 14.8% | 15.3% | 15.1% | 13.2% | 9.9% | 6.2% | 3.5% | 1.6% | 0.6% |
| Rhodosporidium | 4.1% | 3.8% | 3.6% | 3.6% | 3.6% | 3.5% | 3.6% | 3.7% | 3.8% | 3.8% | 3.9% | 3.9% | 3.8% | 3.5% | 2.8% | 2.1% | 1.4% | 0.8% | 0.4% | 0.2% | 0.2% | 0.4% |
| Rhodotorula | 4.1% | 2.4% | 3.1% | 3.6% | 3.8% | 3.9% | 4.1% | 4.1% | 4.0% | 3.6% | 3.0% | 2.3% | 1.6% | 1.0% | 0.6% | 0.3% | 0.2% | 0.1% | 0.1% | 0.0% | 0.0% | 0.0% |
| Filobasidiella | 4.7% | 5.7% | 7.1% | 8.2% | 8.7% | 9.0% | 9.3% | 9.7% | 10.3% | 11.2% | 12.5% | 14.5% | 17.5% | 21.5% | 26.5% | 32.1% | 39.2% | 48.7% | 60.8% | 74.0% | 83.6% | 87.3% |
| Cryptococcus | 4.9% | 3.4% | 4.2% | 4.8% | 5.0% | 5.1% | 5.0% | 4.8% | 4.6% | 4.6% | 4.7% | 5.2% | 5.9% | 6.6% | 7.2% | 7.1% | 6.3% | 4.7% | 3.1% | 2.5% | 2.5% | 2.7% |
| Mucor | 3.4% | 2.1% | 2.5% | 2.8% | 2.9% | 3.0% | 3.0% | 2.8% | 2.6% | 2.4% | 2.1% | 1.6% | 1.2% | 0.7% | 0.4% | 0.3% | 0.2% | 0.2% | 0.1% | 0.1% | 0.1% | 0.0% |
| Rhizopus | 3.6% | 3.3% | 3.8% | 3.8% | 3.5% | 2.9% | 1.9% | 1.2% | 0.7% | 0.5% | 0.4% | 0.3% | 0.2% | 0.2% | 0.2% | 0.1% | 0.1% | 0.0% | 0.0% | 0.0% | 0.1% | 0.0% |
| Ascomycota;Other | 0.0% | 14.5% | 6.8% | 1.5% | 0.5% | 0.3% | 0.2% | 0.2% | 0.1% | 0.1% | 0.1% | 0.1% | 0.1% | 0.1% | 0.2% | 0.2% | 0.2% | 0.2% | 0.2% | 0.2% | 0.2% | 0.6% |
| Fungi;Other | 0.0% | 9.2% | 3.4% | 0.6% | 0.1% | 0.0% | 0.0% | 0.0% | 0.0% | 0.0% | 0.0% | 0.0% | 0.0% | 0.0% | 0.0% | 0.0% | 0.0% | 0.1% | 0.1% | 0.1% | 0.2% | 0.4% |
| Basidiomycota;Other | 0.0% | 2.8% | 1.1% | 0.3% | 0.1% | 0.0% | 0.0% | 0.0% | 0.0% | 0.0% | 0.0% | 0.0% | 0.0% | 0.0% | 0.0% | 0.0% | 0.0% | 0.1% | 0.1% | 0.1% | 0.1% | 0.0% |

# B

| Taxon | mock | MQV10 | MQV11 | MQV12 | MQV13 | MQV14 | MQV15 | MQV16 | MQV17 | MQV18 | MQV19 | MQV20 | MQV21 | MQV22 | MQV23 | MQV24 | MQV25 | MQV26 | MQV27 | MQV28 | MQV29 | MQV30 |
|---|---|---|---|---|---|---|---|---|---|---|---|---|---|---|---|---|---|---|---|---|---|---|
| Acremonium | 4.1% | 4.4% | 4.4% | 4.4% | 4.4% | 4.4% | 4.4% | 4.4% | 4.4% | 4.4% | 4.4% | 4.4% | 4.4% | 4.3% | 4.3% | 4.3% | 4.3% | 4.4% | 4.4% | 4.4% | 4.4% | 4.4% |
| Cladosporium | 8.2% | 9.4% | 9.4% | 9.4% | 9.4% | 9.4% | 9.4% | 9.4% | 9.4% | 9.4% | 9.4% | 9.4% | 9.4% | 9.4% | 9.4% | 9.5% | 9.5% | 9.5% | 9.6% | 9.6% | 9.7% | 9.7% |
| Aspergillus | 14.5% | 11.0% | 11.0% | 11.0% | 11.0% | 11.0% | 11.0% | 11.0% | 11.0% | 11.0% | 11.0% | 11.0% | 11.0% | 11.0% | 11.0% | 11.0% | 10.9% | 10.9% | 10.8% | 10.7% | 10.7% | 10.6% |
| Penicillium | 15.7% | 13.3% | 13.3% | 13.3% | 13.3% | 13.3% | 13.3% | 13.3% | 13.3% | 13.3% | 13.3% | 13.3% | 13.3% | 13.3% | 13.3% | 13.2% | 13.1% | 13.0% | 12.8% | 12.7% | 12.6% | 12.5% |
| Saccharomyces | 2.3% | 1.5% | 1.5% | 1.5% | 1.5% | 1.5% | 1.5% | 1.5% | 1.5% | 1.5% | 1.5% | 1.5% | 1.5% | 1.5% | 1.4% | 1.4% | 1.3% | 1.3% | 1.2% | 1.2% | 1.1% | 1.1% |
| Candida | 12.6% | 14.4% | 14.4% | 14.4% | 14.4% | 14.4% | 14.4% | 14.4% | 14.4% | 14.4% | 14.4% | 14.4% | 14.4% | 14.4% | 14.5% | 14.5% | 14.6% | 14.7% | 14.8% | 14.9% | 15.1% | 15.2% |
| Nakaseomyces | 2.1% | 0.0% | 0.0% | 0.0% | 0.0% | 0.0% | 0.0% | 0.0% | 0.0% | 0.0% | 0.0% | 0.0% | 0.0% | 0.0% | 0.0% | 0.0% | 0.0% | 0.0% | 0.0% | 0.0% | 0.0% | 0.0% |
| Trichoderma | 7.4% | 7.2% | 7.2% | 7.2% | 7.2% | 7.2% | 7.2% | 7.2% | 7.2% | 7.2% | 7.2% | 7.2% | 7.2% | 7.2% | 7.2% | 7.1% | 7.1% | 7.0% | 7.0% | 6.9% | 6.9% | 6.8% |
| Fusarium | 8.4% | 10.6% | 10.6% | 10.6% | 10.6% | 10.6% | 10.6% | 10.6% | 10.6% | 10.6% | 10.6% | 10.6% | 10.7% | 10.7% | 10.7% | 10.7% | 10.7% | 10.7% | 10.7% | 10.7% | 10.7% | 10.7% |
| Rhodosporidium | 4.1% | 4.6% | 4.6% | 4.6% | 4.6% | 4.6% | 4.6% | 4.6% | 4.6% | 4.6% | 4.6% | 4.6% | 4.6% | 4.6% | 4.6% | 4.6% | 4.6% | 4.7% | 4.7% | 4.7% | 4.8% | 4.8% |
| Rhodotorula | 4.1% | 4.2% | 4.2% | 4.2% | 4.2% | 4.2% | 4.2% | 4.2% | 4.2% | 4.2% | 4.2% | 4.2% | 4.2% | 4.2% | 4.2% | 4.2% | 4.3% | 4.3% | 4.3% | 4.4% | 4.4% | 4.4% |
| Filobasidiella | 4.7% | 5.2% | 5.2% | 5.2% | 5.2% | 5.2% | 5.2% | 5.2% | 5.2% | 5.2% | 5.2% | 5.2% | 5.2% | 5.2% | 5.2% | 5.3% | 5.3% | 5.3% | 5.4% | 5.5% | 5.5% | 5.6% |
| Cryptococcus | 4.9% | 6.0% | 6.0% | 6.0% | 6.0% | 6.0% | 6.0% | 6.0% | 6.0% | 6.0% | 6.0% | 6.0% | 6.0% | 6.0% | 6.0% | 6.0% | 6.1% | 6.1% | 6.2% | 6.3% | 6.3% | 6.4% |
| Mucor | 3.4% | 3.1% | 3.1% | 3.1% | 3.1% | 3.1% | 3.1% | 3.1% | 3.1% | 3.1% | 3.1% | 3.1% | 3.1% | 3.1% | 3.1% | 3.1% | 3.1% | 3.1% | 3.0% | 3.0% | 3.0% | 3.0% |
| Rhizopus | 3.6% | 5.0% | 5.0% | 5.0% | 5.0% | 5.0% | 5.0% | 5.0% | 5.0% | 5.0% | 5.0% | 5.0% | 5.0% | 5.0% | 5.0% | 5.1% | 5.1% | 5.0% | 5.0% | 4.9% | 4.9% | 4.8% |

# C

| Taxon | **mock** | ROI02 | ROI03 | ROI04 | ROI05 | ROI06 | ROI07 | ROI08 | ROI09 | ROI10 | ROI11 | ROI12 |
|---|---|---|---|---|---|---|---|---|---|---|---|---|
| Acremonium | 4.1% | 4.0% | 4.1% | 4.1% | 4.1% | 4.1% | 4.2% | 4.1% | 4.1% | 4.1% | 4.0% | 4.1% |
| Cladosporium | 8.2% | 7.6% | 7.8% | 7.9% | 8.0% | 8.1% | 8.1% | 8.1% | 8.1% | 8.1% | 8.2% | 8.2% |
| Aspergillus | 14.5% | 13.7% | 14.0% | 14.1% | 14.1% | 14.1% | 14.1% | 14.0% | 14.0% | 13.9% | 13.9% | 13.9% |
| Penicillium | 15.7% | 14.4% | 14.6% | 14.8% | 14.8% | 14.8% | 14.8% | 14.8% | 14.7% | 14.6% | 14.5% | 14.4% |
| Saccharomyces | 2.3% | 1.0% | 1.0% | 1.0% | 1.0% | 0.9% | 0.9% | 0.9% | 0.9% | 0.8% | 0.8% | 0.8% |
| Candida | 12.6% | 13.2% | 13.6% | 13.8% | 14.0% | 14.2% | 14.3% | 14.4% | 14.5% | 14.6% | 14.7% | 14.8% |
| Nakaseomyces | 2.1% | 0.4% | 0.4% | 0.4% | 0.4% | 0.4% | 0.4% | 0.4% | 0.3% | 0.3% | 0.3% | 0.3% |
| Trichoderma | 7.4% | 5.3% | 5.5% | 5.6% | 5.7% | 5.7% | 5.6% | 5.6% | 5.6% | 5.6% | 5.6% | 5.6% |
| Fusarium | 8.4% | 8.7% | 8.9% | 9.0% | 9.0% | 9.0% | 9.1% | 9.1% | 9.1% | 9.2% | 9.3% | 9.3% |
| Rhodosporidium | 4.1% | 4.0% | 4.1% | 4.2% | 4.2% | 4.2% | 4.2% | 4.2% | 4.2% | 4.3% | 4.3% | 4.2% |
| Rhodotorula | 4.1% | 3.4% | 3.5% | 3.7% | 3.7% | 3.8% | 3.9% | 3.9% | 4.0% | 4.0% | 4.0% | 4.1% |
| Filobasidiella | 4.7% | 4.8% | 5.0% | 5.1% | 5.2% | 5.3% | 5.4% | 5.5% | 5.6% | 5.6% | 5.7% | 5.8% |
| Cryptococcus | 4.9% | 5.5% | 5.7% | 5.8% | 5.9% | 6.0% | 6.1% | 6.2% | 6.2% | 6.3% | 6.4% | 6.5% |
| Mucor | 3.4% | 2.7% | 2.7% | 2.7% | 2.7% | 2.7% | 2.7% | 2.8% | 2.7% | 2.8% | 2.8% | 2.8% |
| Rhizopus | 3.6% | 3.9% | 3.9% | 3.9% | 4.0% | 3.9% | 3.9% | 3.9% | 3.9% | 3.9% | 3.9% | 4.0% |
| Ascomycota;Other | 0.0% | 1.7% | 1.1% | 0.8% | 0.6% | 0.5% | 0.5% | 0.4% | 0.4% | 0.4% | 0.3% | 0.3% |
| Fungi;Other | 0.0% | 1.5% | 0.9% | 0.6% | 0.5% | 0.4% | 0.4% | 0.3% | 0.3% | 0.3% | 0.2% | 0.2% |

**Fig. S4. Mock community mycobiota analyses using three sequencing platforms.**

Percent relative abundance of major fungal genera in the mock community obtained from the results generated by IonPGM (A), MiSeq (B), and PacBio (C). (A, B) Read-trimming was carried out using an ideal relative abundance of the mock community and MQV 10 – 30. Relative abundance of each of the major fungal genera are shown as a bar graph. (C) Consensus sequences were created with the ideal values for the mock community and between 2 and 12 full passes. Relative abundance of each of the major fungal genera is shown as a bar graph.
